# Bridging priorities: Stakeholder preferences, networks, and barriers in road-stream crossing management

**DOI:** 10.1371/journal.pone.0339740

**Published:** 2026-01-05

**Authors:** Koorosh Asadifakhr, Jingyan Huang, Pauline Perkins, Kevin Lucey, Haiying Wang, Fei Han, Weiwei Mo

**Affiliations:** 1 Department of Civil and Environmental Engineering, University of New Hampshire, Durham, New Hampshire, United States of America; 2 Natural Resources & Earth Systems Science Program, University of New Hampshire, Durham, New Hampshire, United States of America; 3 New Hampshire Department of Environmental Services, Concord, New Hampshire, United States of America; 4 Department of Statistics, University of Connecticut, Storrs, Connecticut, United States of America; Bangladesh Council of Scientific and Industrial Research, BANGLADESH

## Abstract

Road-stream crossings (RSCs) represent a critical nexus of infrastructure resilience and ecosystem health, yet fragmented governance and institutional silos hinder effective management. This study used a co-produced survey to assess stakeholder priorities, map the stakeholder collaboration network, and characterize non-financial barriers to RSC decision-making in New Hampshire, USA. Analyses included the Kruskal–Wallis and Dwass–Steel–Critchlow–Fligner tests to evaluate differences in priorities across stakeholder groups, social network analysis to identify central actors, and inductive content analysis for non-financial challenges. Flood vulnerability was the most widely supported goal, offering common ground for collaboration. However, divergences in wildlife conservation, environmental quality, structural risk, and road criticality highlighted persistent tensions between conservation and transportation stakeholders. Socioeconomic goals, including economic impact and environmental justice, received lower ratings and minimal divergence, indicating systemic neglect rather than conflict. Social network analysis identified the New Hampshire Departments of Transportation and Environmental Services as central actors, enabling coordination but concentrating decision power. Content analysis revealed key non-financial barriers: lack of prioritization, project complexity, regulatory burdens, and limited municipal capacity. These findings highlight opportunities for inclusive, multi-benefit decision frameworks, regulatory streamlining, and investments in local technical capacity to better align infrastructure planning with ecological and community needs.

## 1. Introduction

Road-stream crossings (RSCs) are critical infrastructure (e.g., culverts, bridges, arches) that enable roads to traverse water bodies. In the United States, there are over six million crossings, many of which are aging or in poor condition [1–3]. These deteriorating structures pose persistent risks of flooding, road failures, fragmenting aquatic habitats, and degrading water quality [4–7]. In response, municipal, regional, and national initiatives have been implemented to identify and/or upgrade high-risk RSCs, including notably, the National Aquatic Barrier Inventory & Prioritization Tool led by the Southeast Aquatic Resources Partnership (SARP), as well as the New Hampshire Stream Crossing Initiative (NHSCI) [2,8–10]. Despite growing awareness and ongoing efforts, current RSC management practices remain largely fragmented [11,12]. Responsibilities and funding are distributed among agencies with distinct mandates, regulatory frameworks, and priorities, resulting in disjointed planning processes and uncoordinated implementation [13–15]. This fragmentation leads to inefficient allocation of resources and missed opportunities to deliver more cost-effective, multi-benefit outcomes that could simultaneously address transportation safety, ecological health, and social equity. At the core of the challenge to joint action is the complexity of governance in aligning priorities, bridging institutional silos, and balancing trade-offs across agencies. A critical first step toward cohesive cross-sectoral RSC management is to clarify what different stakeholders value, how those values align or conflict, and which structural barriers inhibit collective action.

Despite the widespread recognition of the need for effective RSC management [11,12], previous RSC assessment/prioritization efforts have predominantly focused on either a single aspect of RSC performance or the interests of a single stakeholder group [4,16–27], such as habitat connectivity [16–23], structural integrity [24], cost-effective design [4,27], or erosion control [25,26]. Only a few studies have incorporated multiple RSC performance evaluation criteria in their analyses [10,11,28,29]. Baker et al. (2001) developed a condition index based on structural (e.g., structure age, scour, corrosion), ecological (e.g., sedimentation, physical blockage), and transportation (e.g., average daily traffic) metrics for RSC management prioritization [28]. Milone and MacBroom (2016) incorporated both transportation (e.g., structural condition, flood vulnerability, geomorphic compatibility) and ecological (e.g., aquatic organism passage) metrics into RSC prioritization. Beyond transportation and ecological considerations, Roy et al. (2020) further added economic cost into their decision matrix. These studies represent important initial efforts towards identifying and balancing the interests of key stakeholders, primarily transportation and environmental agencies. However, the spectrum of RSC stakeholders extends well beyond these two sectors. Development of holistic RSC management plans requires a comprehensive and methodologically consistent assessment of the interests and priorities of all relevant stakeholders. Such an approach can facilitate the identification of shared objectives and support more strategic use of constrained funding to achieve broad, cross-sector benefits.

Beyond capturing and understanding stakeholder priorities and interests, effective RSC management also requires a thorough understanding of the key actors in the current RSC stakeholder network. This will support the identification of potential coordinators/facilitators to enhance collaborative RSC decision-making, ensure successful project development and completion over sometimes decades-long project timelines, and balance diverse stakeholder interests. Nevertheless, our understanding of the extent and nature of interactions and collaborations among various RSC stakeholders and their influence on RSC management decisions remains extremely limited. Of the few previous studies that have investigated RSC stakeholder decision-making and collaboration, Andrew-Nielsen (2023) analyzed how stakeholders in Northern Sweden incorporate sustainability concepts into RSC management practices [30], while Kinder (2017) employed social network analysis to explore the social capital structure and dynamics in stream restoration in the Upper Shavers Fork of West Virginia [31]. Whereas Kinder’s work is most closely aligned with stakeholder network analysis, it centers on a single stream restoration project rather than offering broader insights into general RSC management practices.

Effective RSC management can also be impeded by challenges that extend beyond financial constraints, such as complex regulatory procedures [32,33], community disruptions as a result of RSC management activities [34], complications related to property ownership [33], the requirement of specialized, technical expertise [35–38], and the lack of comprehensive RSC condition data [10–12,29]. Although these challenges have been individually recognized, there is an absence of integrated, stakeholder-inclusive assessments of such challenges to improve future RSC management practices.

To address these, this study used a stakeholder co-produced survey instrument to systematically elicit RSC stakeholder priorities and interests, capture the RSC stakeholder network, and identify key non-financial RSC management challenges across the State of New Hampshire, USA. The differences of RSC stakeholder priorities and interests were then assessed using the Kruskal–Wallis (KW) test and the Dwass–Steel–Critchlow–Fligner (DSCF) post hoc test, while the stakeholder decision network was analyzed using social network analysis (SNA). Last but not least, we applied content analysis to synthesize and evaluate stakeholder-perceived non-financial RSC management challenges. Together, this study seeks to answer three main research questions: 1) What are the key stakeholder priorities in RSC management, and how do priorities vary among different stakeholder groups? 2) Who are the central actors in the New Hampshire RSC stakeholder network? 3) What are the main non-financial challenges to RSC management?

## 2. Methods

This section describes the design and administration of the survey instrument used to gather stakeholder insights (Section 2.1), the statistical methods applied to analyze and interpret stakeholder preferences (Section 2.2), the social network analysis applied to identify key actors within the New Hampshire RSC stakeholder network (Section 2.3), and the content analysis conducted to explore the non-financial RSC management challenges (Section 2.4).

### 2.1. Survey Design and Administration

To ensure that the survey design for RSC prioritization accurately reflected the needs, priorities, and operational realities of practitioners, we established a Technical Advisory Committee (TAC) for survey co-development. The TAC was composed of over 20 stakeholders from organizations that routinely make or influence decisions related to RSCs, including the New Hampshire Department of Transportation (NHDOT), New Hampshire Department of Environmental Services (NHDES), New Hampshire Fish and Game Department (NHF&G), The Nature Conservancy (TNC), various Regional Planning Commissions (RPCs), and the private sector. These stakeholders collectively represent expertise in transportation infrastructure, environmental regulation, ecological conservation, and local planning, thereby ensuring a broad representation of relevant stakeholder perspectives. Instead of building this group from scratch, we leveraged the long-standing network of the NHSCI, which had already fostered collaboration among many of these stakeholders. The partnership with the NHSCI allowed for rapid engagement and trust-based coordination. Additional members from RPCs, TNC, and the private sector were intentionally included to ensure that both local planning and non-profit perspectives, which are often underrepresented in state-level RSC planning, were directly involved. The TAC played a central role in the iterative development of the survey instrument. The process was organized around three structured meetings, each serving a distinct purpose in shaping the content, structure, and dissemination strategy of the survey.

- **Initiation Meeting:** The project overview was presented to the NHSCI, including the project objectives, existing literature on RSC management [9,10,28,29,39–44], and the proposed survey methodology. This meeting served as a grounding session to establish a common understanding of the project scope and ensure alignment among all participants.
- **TAC Meeting 1:** An initial set of overarching RSC management goals and associated evaluation criteria were presented, along with a draft list of intended survey recipients. TAC members were invited to criticize and refine both the substance of the goals and the technical clarity of the evaluation criteria. The team documented each agency’s existing RSC-related interests and priorities to identify commonalities and ensure that the distinct priorities were represented. Feedback was collected during the meeting through open discussion and was supplemented by written comments submitted afterward. In cases where further feedback was needed, follow-up one-on-one meetings were held with individual TAC members.
- **TAC Meeting 2:** This meeting presented a revised version of the survey that incorporated the feedback from TAC Meeting 1. The focus of this session was to finalize the survey structure, clarify any remaining ambiguities in language, and ensure the evaluation criteria were meaningful and interpretable across sectors. Additionally, the TAC also discussed and finalized strategies for distributing the survey in a way that would maximize participation across disciplines and regions.

In the finalized survey, participants were presented with eight overarching goals for RSC prioritization along with their definitions (Table 1). They were asked to rate the importance of each goal using a six-point ordinal scale: “Not at all important,” “Slightly important,” “Moderately important,” “Very important,” “Extremely important,” and “Unfamiliar.” An open-ended question invited participants to suggest additional goals not included in the survey. After assessing the goals, participants were shown the evaluation criteria associated with each goal along with their definitions/explanations (Table 1; see the Supporting Information (S1 File) for the detailed evaluation criteria for each goal and their explanations). Each criterion was rated using the same six-point ordinal scale, with an additional response option: “Not related to the goal” for criteria deemed irrelevant. Participants could also propose new criteria not included in the survey. The final section of the survey collected qualitative input on broader aspects related to RSC management, including interagency collaboration opportunities and non-financial barriers.

**Table 1:** Overarching goals related to RSC management, their definitions, and example evaluation criteria under each goal.

Overarching Goals	Definitions	Example Evaluation Criteria under the Goal
Wildlife Conservation and Restoration	The ability of fish and wildlife to move through an RSC structure and the potential to restore habitat connectivity within the riverine system.	Aquatic Organism Passage, Habitat Quality, Special Species Presence and Population, Terrestrial Organism Passage, Risk of Wildlife Collision
Environmental Quality	The current water quality of the stream segment and the extent to which the RSC structure influences water quality.	Erosion, Water Quality Impairment, Water Use
Road Criticality	The significance of the road segment and RSC structure in maintaining the functional operation of the transportation system.	Annual Average Daily Traffic, Distance to the location of Important Services, Road Tier, Detour Length
Economic Impact	The short- and long-term financial implications of replacing a stream crossing structure.	Capital Cost, Operation and Maintenance, Long-Term Economic Benefits, Repaving Schedule
Flood Vulnerability	The extent to which an RSC accommodates the natural shape of the river, facilitates flood flow transport, and mitigates flood risk.	Hydraulic Capacity, Documented History of Flooding, Climate Resiliency
Structural Risk	The physical condition of the RSC structure and other factors affecting its structural integrity, risk, and potential impact.	Structural Condition, Size and Depth of Cover, Material, Age
Community Support and Readiness	The community’s preparedness for and commitment to RSC management.	Funding Availability and Accessibility, State and Federal Support, Community Preparedness
Environmental Justice	Ensuring the fair distribution of resources and benefits across communities (distributional equity) while promoting inclusive and transparent decision-making processes (procedural equity).	Procedural Equity, Distributional Equity

**Fig 1:**
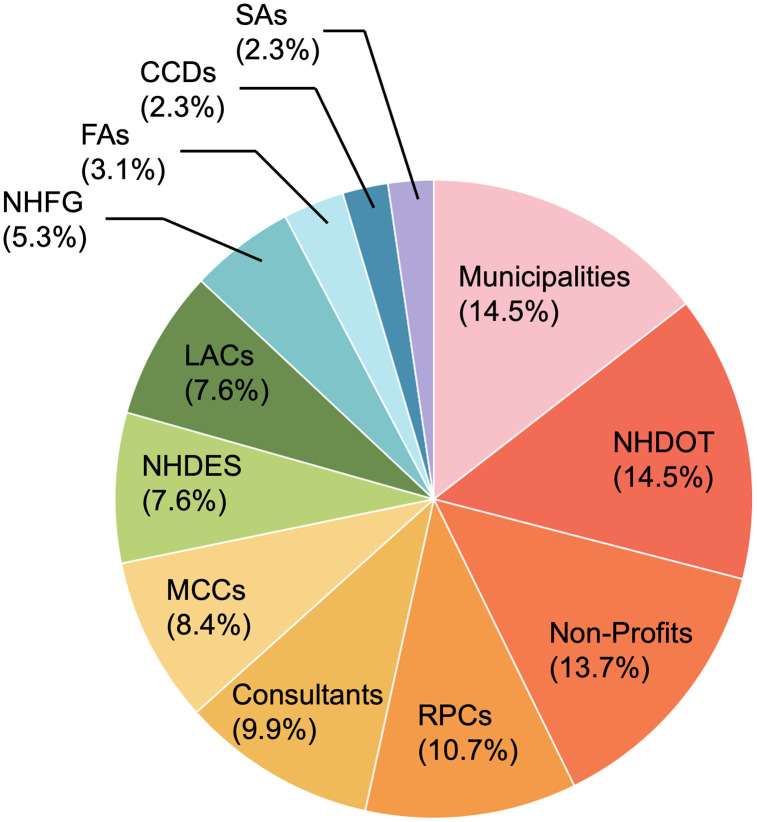
Distribution of survey participants by agency type. Chart shows the percentage of total validated survey responses (N = 130) grouped into 12 stakeholder categories: 1) NHDOT; 2) NHDES; 3) NHFG; 4) Local advisory committee (LACs); 5) RPCs; 6) Engineering consulting firms (Consultants); 7) Municipalities; 8) County conservation districts (CCDs); 9) Municipal conservation commissions (MCCs); 10) Non-profit organizations (Non-profits); 11) Federal agencies (FAs) including National Oceanic and Atmospheric Administration (NOAA), **U.**S. Fish and Wildlife Service (USFWS), and Natural Resources Conservation Service (NRCS); 12) Other state agencies (SAs), including NH Homeland Security and Emergency Management (NHHSEM), NH Department of Agriculture (NHDA), and NH Department of Business and Economic Affairs (NHDBEA).

The co-designed survey instrument was administered via Qualtrics, and the project was approved by the Institutional Review Board for the Protection of Human Subjects in Research at the University of New Hampshire (IRB-FY2023–154). Written informed consent was obtained from all participants prior to their participation in the survey. To broaden survey participation and ensure geographic and disciplinary coverage, a multi-pronged outreach strategy was implemented prior to the survey launch, involving six in-person meetings, twelve webinars, two newsletters, and conference presentations (Table S3 in the S1 File). After the outreach, the survey was distributed via email to 211 identified stakeholders, who were encouraged to forward it to any other potentially interested parties across the State of New Hampshire between March 25 and May 15, 2024. The survey received 139 distinct responses within the period with at least one question answered, yielding an overall response rate of 65.9%. From these, we excluded 9 responses that lacked agency information from the following analyses, as this omission prevented us from verifying the respondents as legitimate stakeholders. The remaining 130 responses were categorized into 12 stakeholder groups based on organizational roles and functions. Fig 1 shows the percentage distribution of survey participants by stakeholder group.

### 2.2. Analysis of stakeholder preferences

Likert-scale ratings of the eight overarching goals were coded from 0 to 4 to represent “Not at all important,” “Slightly important,” “Moderately important,” “Very important,” and “Extremely important,” respectively. “Unfamiliar” was excluded from the analysis because it does not represent a value judgment, and its inclusion would compromise the comparability of ranked responses across groups. Ratings of the evaluation criteria associated with each goal were coded in the same manner, except that responses marked “Not related to the goal” were treated as equivalent to “Not at all important,” as both indicate that the item is not a relevant factor in the respondent’s decision-making. Furthermore, if all associated criteria for a goal were marked as “Not related to the goal” in a survey response, these ratings were excluded to avoid skewing the results. Since all questions were optional for respondents, unanswered questions were retained in the dataset and treated as missing data for analysis. For each goal and criterion, a relative importance index (RII) was calculated using Equation 1 to reflect the overall importance.


$${\text{RII}}_{i}=\frac{\sum_{j=\text{0}}^{A}w_{j}f_{ij}}{A\times N_{i}}$$ (1)

Where *i* is the goal and criterion index; *w*_*j*_ is the numerical weight assigned to the *j*th response option on the Likert scale (0 for “Not at all important” through 4 for “Extremely important”); *f*_*ij*_ is the number of respondents who selected the *j* th response option for Item *i*; *A* is the maximum possible weight on the response scale (4 in this study); and *N*_*i*_ is the total number of respondents who rated Item *i*.

To determine whether significant differences existed among the 12 stakeholder groups in their prioritization of the overarching goals and associated evaluation criteria, two non-parametric statistical methods were employed: the KW test (using the *scipy.stats* library in Python [45,46]) and the DSCF post hoc test (using the *NSM3* package in R [47]). The KW test examined the overall differences across all stakeholder groups, while the DSCF test identified specific group pairs with significant differences when the KW test indicated an overall significance (*p* < 0.05). These methods were selected for their suitability in analyzing ordinal, ranked data without requiring the assumption of a normal distribution [48,49]. The analysis was applied to all questions related to overarching goals and evaluation criteria. For each KW test, we report the H statistic, which measures how different the ranked responses are across groups, and the corresponding p-value, which indicates whether those differences are statistically significant. The H value is calculated first based on the data, and the p-value is then derived from the H value. In general, a larger H value reflects greater differences among group responses (assuming relatively balanced group sizes and response counts) and is typically associated with a smaller p-value, suggesting the observed differences are unlikely due to chance. For DSCF results, we report the absolute values of the standardized W statistic (|*W*|) and the adjusted *p*-values. Similarly, larger |*W*| values suggest more pronounced pairwise differences, provided that group sizes are reasonably balanced.

### 2.3. Social network analysis of stakeholder collaborations

We used SNA to identify key actors within the RSC stakeholder decision network and to map patterns of collaboration, providing insights into governance structure and informing future coordination efforts. In this study, the network was constructed from respondents’ answers to a survey question asking them to identify organizations they collaborate with on RSC-related projects. Each organization was represented as a node, and each reported collaboration between two organizations was mapped as an edge connecting two nodes. Expanding on the stakeholder groups defined in Section 2.1, agencies with distinct mandates (e.g., NHDOT, NHDES, NHFG) were treated as individual nodes, while organizations with similar roles (e.g., RPCs, municipalities) were aggregated into single representative nodes. The final network consisted of 30 nodes and 103 edges, modeled as undirected and unweighted based on the assumption that all reported collaborations are reciprocal and equal in importance. A full list of included nodes and their corresponding organizations is provided in Table S2 of the S1 File. We used *NetworkX*, a Python-based library [50], to model the network and calculate degree centrality scores, defined as the number of direct collaborative links associated with each stakeholder. Higher centrality scores were interpreted as indicators of greater collaborative involvement, influence, or leadership within the RSC governance structure.

### 2.4. Content Analysis of Non-Financial Challenges

To identify and categorize non-financial challenges in RSC management, we employed inductive content analysis of responses from a survey question asking participants to describe such challenges. This method is well-suited for deriving meaningful themes from unstructured qualitative data without imposing predefined categories [51]. In the initial coding stage, recurring concepts were first manually labeled with descriptive codes (e.g., “permitting,” “staff shortages,” “coordination issues”). These codes were then grouped into broader thematic categories based on shared meanings. For instance, “permitting,” “design requirements,” and “historic considerations” were combined under the theme “Regulatory Barriers,” reflecting challenges related to regulatory processes and compliance. The coding and theme development processes were iterative, with themes refined over multiple rounds of review to ensure accuracy and completeness. Table 2 summarizes the final set of themes, their descriptions, and associated codes. After coding the data, the content analysis followed a structured process, in which we first quantified the frequency of each identified theme and associated code to determine the most prevalent barriers. We then examined the distribution of themes across stakeholder groups to identify agency-specific patterns or concerns. The coding and theme development processes were iterative, with themes refined over multiple rounds of review to ensure accuracy and completeness.

**Table 2:** Themes and codes for content analysis.

Themes	Description	Codes
Land Ownership	Issues associated with land rights and ownership disputes.	Land ownership
Regulatory Barriers	Challenges with permitting, design standards, historical considerations, and lack of regulatory enforcement.	Permitting
		Historic consideration
		Design requirements
		Lack of regulation-enforcement
Capacity	Limitations in organizational resources include insufficient staffing levels, lack of specialized technical expertise, and inadequate availability of contractors.	Staff
		Expertise
		Contractor availability
Lack of Prioritization	Absence of a system-level, consistent, and multi-criteria prioritization framework, balancing conflicting stakeholder interests.	System-level prioritization
		Conflicting interests
		Consistent prioritization
		Multi-criteria prioritization
		Prioritization framework
Coordination Issues	Challenges in achieving effective collaboration among stakeholders include inefficient inter-agency communication, difficulties in securing stakeholder buy-in, and broader coordination challenges.	Buy-in coordination
		Inter-agency coordination
		General coordination issues
Public Education	Lack of public awareness and education on the benefits of RSC replacement projects.	Education
		Awareness
Project Complexity	Technical, logistical, and operational challenges involved in replacement projects.	Construction: traffic control
		Design
		Time: project delivery
		Permitting
Leadership	Influence of leadership and decision-making on the direction and execution of replacement projects.	Leadership
Lack of Data	Gaps in data quality, availability, or accessibility for informed decision-making.	Age
		Aquatic passage for dams, waterfalls, and fish ladders
		Climate resiliency
		Prioritization data
		Condition

## 3. Results and discussion

### 3.1. Stakeholder preferences in RSC management

**Fig 2:**
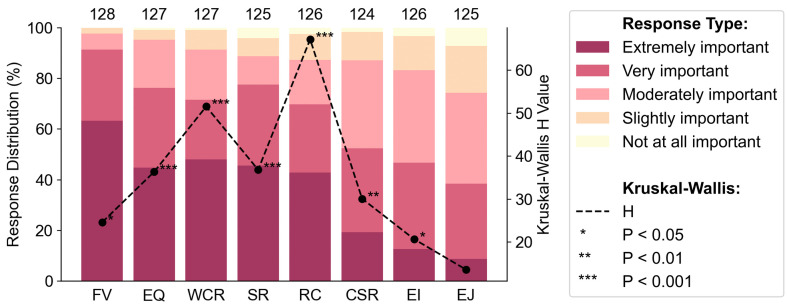
Distribution of stakeholder responses regarding the perceived importance of eight overarching goals in road-stream crossing (RSC) prioritization, displayed as stacked bar charts. Stacked bars show the percentage of respondents selecting each response category, from “Extremely important” to “Not at all important,” for each goal: Flood Vulnerability (FV), Wildlife Conservation and Restoration (WCR), Environmental Quality (EQ), Structural Risk (SR), Road Criticality (RC), Community Support and Readiness (CSR), Economic Impact (EI), and Environmental Justice (EJ), shown on the horizontal axis. Each rating represents one survey response from the 130 validated respondents across 12 stakeholder groups. The total number of non-missing responses (N) for each goal is shown above the corresponding bar. The Kruskal-Wallis (KW) test H-values (right vertical axis), represented by black dots connected by a dashed line, indicate the degree of variation in responses across stakeholder groups. Statistical significance is denoted by asterisks (* p < 0.05, ** p < 0.01, *** p < 0.001).

While preference analyses were conducted for both the overarching goals and their associated evaluation criteria, we focus here primarily on the findings related to the overarching goals. Results pertaining to the detailed evaluation criteria are provided in the S1 File. Of the eight overarching goals, Flood Vulnerability (FV) received the highest importance rating (RII = 0.88) across all stakeholder groups, followed by Environmental Quality (EQ, RII = 0.78), Wildlife Conservation and Restoration (WCR, RII = 0.77), Structural Risk (SR, RII = 0.76), and Road Criticality (RC, RII = 0.74) (Fig 2). The high FV rating by stakeholders is likely tied to the potential of RSCs to obstruct flow and cause overtopping, which can pose significant public safety risks to traffic, nearby housing, and surrounding communities [52]. Emergency replacements of RSCs have been reported to cost 4–140 times more than planned replacements, primarily due to traffic disruptions and logistical complexities [53]. The relatively high ratings for EQ, WCR, SR, and RC reflect the strong stakeholder motivation in sustaining ecosystem connectivity, water quality, infrastructure reliability, and transportation network functionality.

In contrast, the socioeconomic goals, Community Support and Readiness (CSR, RII = 0.64), Economic Impact (EI, RII = 0.6), and Environmental Justice (EJ, RII = 0.53) received lower ratings (Fig 2), suggesting a limited sense of urgency among stakeholders regarding these goals despite their long-term community implications. This is likely because formal RSC prioritization frameworks often lack standardized socio-economic criteria, leading project selection to default to technical and regulatory factors, while community-oriented objectives are treated as secondary. Stakeholders may also be less familiar with or informed about these socio-economic goals, further contributing to their lower rankings. Moreover, the widespread absence of measurable indicators for EJ can be the reason for its persistently low ratings. This is especially concerning, given that underrepresented communities often face disproportionate risks from aging and failing infrastructure and extreme weather events [54,55]. Addressing this issue may require dedicated funding for projects that benefit vulnerable populations, meaningful community engagement early in the planning process, and the incorporation of cost-benefit indicators that extend beyond purely economic metrics.

The KW results indicate a high level of agreement among stakeholder groups on the importance of FV, reflecting strong and aligned interests that could serve as a strategic entry point for stakeholder collaboration and policy innovation. Significant stakeholder disagreements (p < 0.001) exist for WCR, EQ, SR, and RC goals despite their relatively high overall importance ratings, with RC showing the highest divergence (H = 67.20, p < 0.001). This indicates that while some stakeholder groups consider these goals highly important, others show limited interest. These areas represent the greatest potential for stakeholder conflict and are where multi-criteria decision-making could be particularly valuable in balancing competing interests and identifying win-win solutions [56]. Lastly, stakeholders agreed on the low importance of the CSR, EI, and EJ goals. Notably, EJ ratings show the least difference across groups (H = 13.62, p = 0.255), reinforcing the concern that equity considerations remain consistently overlooked in RSC planning.

**Fig 3:**
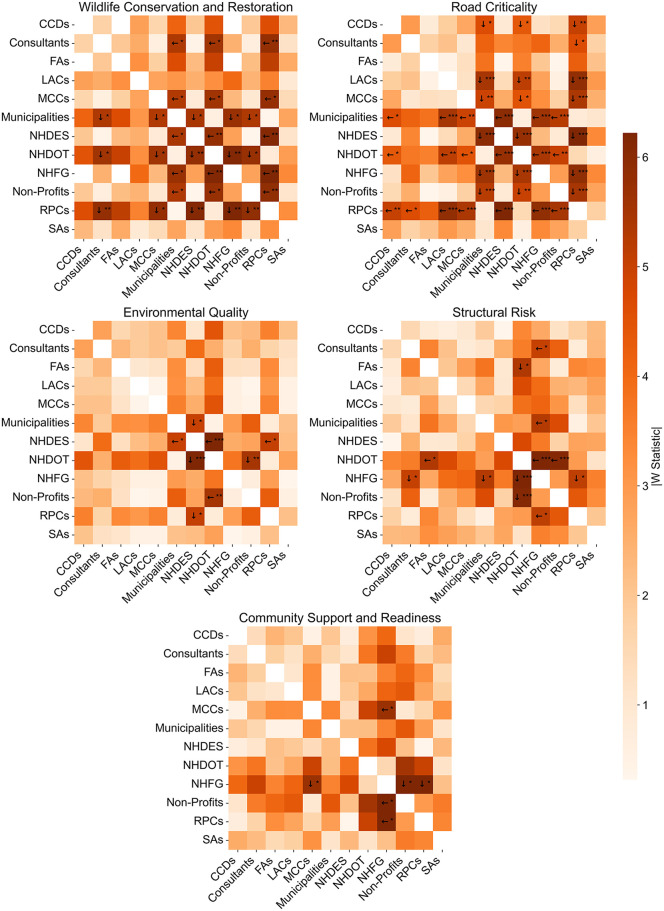
Pairwise stakeholder differences in road-stream crossing (RSC) prioritization, based on the Dwass-Steel-Critchlow-Fligner (DSCF) post hoc test for overarching goals with significant Kruskal-Wallis results. The matrix displays the absolute value of the DSCF test statistic (|W|) for each pair of stakeholder groups; darker shading indicates greater differences in goal ratings. Arrows indicate the direction of higher median ratings, identifying which group assigned greater importance to the goal. Asterisks denote statistically significant pairwise differences after adjustment for multiple comparisons (* adjusted p < 0.05, ** adjusted p < 0.01, *** adjusted p < 0.001). Each cell is based on the set of individual Likert scale responses for the corresponding two stakeholder groups.

A deeper dive into the pairwise stakeholder differences through the DSCF post doc test (Fig 3) reveals that the most pronounced pairwise disagreements occur for WCR and RC goals, especially between transportation-oriented stakeholders (e.g., NHDOT, RPCs, and municipalities) and conservation-oriented stakeholders (e.g., NHFG, NHDES, and environmental non-profits). These differences reflect contrasting institutional missions: transportation-oriented stakeholders, such as RPCs and NHDOT, prioritize roadway functionality, connectivity, and asset management, whereas conservation-oriented stakeholders, like NHFG, emphasize habitat restoration, ecological connectivity, and species protection. Funding structures also shape the differences. Transportation agencies typically receive state and federal transportation funding earmarked for safety and mobility improvements, while conservation agencies rely heavily on revenue from hunting and fishing licenses and federal wildlife grants, which prioritize ecological outcomes. These divisions highlight a persistent governance challenge in RSC management: reconciling infrastructure performance with environmental integrity. Cross-agency task forces and science-based trade-off modeling tools may help bridge the gap by facilitating co-developed solutions that reflect both ecological and transportation priorities.

### 3.2. Stakeholder Network Structure and Collaborative Governance in RSC Management

**Fig 4:**
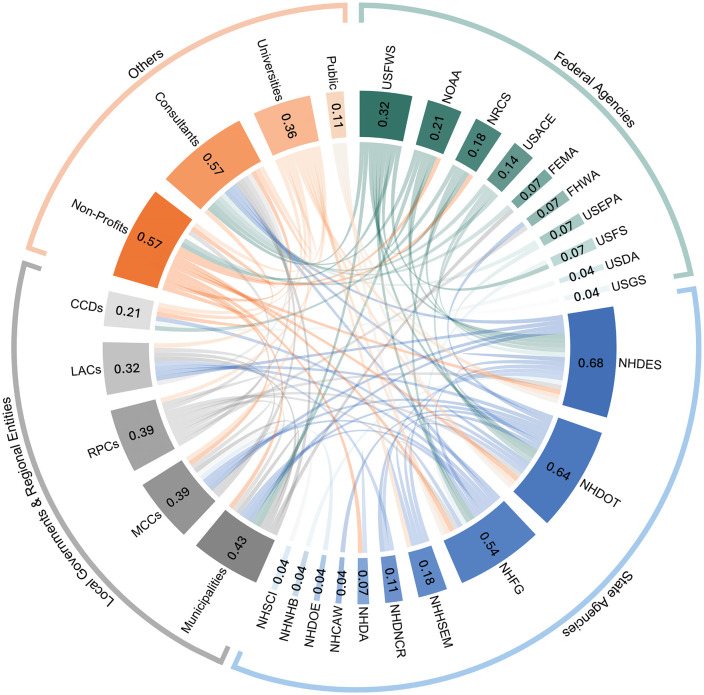
Stakeholder collaboration network in road stream crossing management in New Hampshire. Each color represents a stakeholder category, and connecting bands represent reported collaborations between them. The numbers within each segment indicate the degree of centrality of stakeholders within the network.

Our SNA analysis shows that the NHDOT and NHDES have the most collaboration links in NH’s RSC stakeholder decision network (Fig 4). Their centrality reflects their institutional responsibilities in RSC management: NHDES regulates RSC environmental compliance and permitting, while NHDOT oversees RSC structural integrity and transportation safety. NHDOT owns and manages a substantial portion of the state’s transportation network, which is closely integrated with municipal roads. This interdependence can create jurisdictional and priority-setting conflicts. For example, a municipality may address flooding on a local road, but full mitigation may require improvements to an intersecting state road, projects over which the municipality has no control, and which may not align with NHDOT’s priorities. Other prominent actors include non-profits, consultants, and NHFG, who contribute to project implementation, support funding and advocacy, and conservation efforts, respectively. Collectively, the aforementioned stakeholders serve as gatekeepers, shaping how RSC projects are initiated, approved, and implemented. While high centrality allows these actors to bridge otherwise disconnected stakeholders and promote information flow, this concentration of influence can create power asymmetries, where a few agencies disproportionately shape decisions about project priorities, timelines, and resource distribution. For example, municipalities must secure permits from NHDES for most RSC replacements, making local project timelines contingent on state regulatory approval. Similarly, projects not included in NHDOT’s Ten-Year Plan often struggle to access state funding, limiting local powers in advancing RSC upgrades. In contrast, peripheral stakeholders, such as CCDs, LACs, and MCCs, exhibited low degree centrality scores (all < 0.2), indicating limited collaboration links within the network. This reinforces their advisory status rather than decision-making authority, which can diminish their influence despite their localized knowledge and site-specific expertise. Because final approvals and funding decisions largely rest with state agencies, their localized knowledge and on-the-ground insights can be overshadowed by broader policy mandates and priorities, risking the omission of local community needs. To avoid such risks, the NHSCI, which already includes most central stakeholders, may be leveraged to establish rotating working groups that deliberately include peripheral stakeholders to help strengthen their network connectivity and ensure that local knowledge is integrated into decision-making processes.

### 3.3. Addressing Non-Financial Challenges in RSC Management

**Fig 5:**
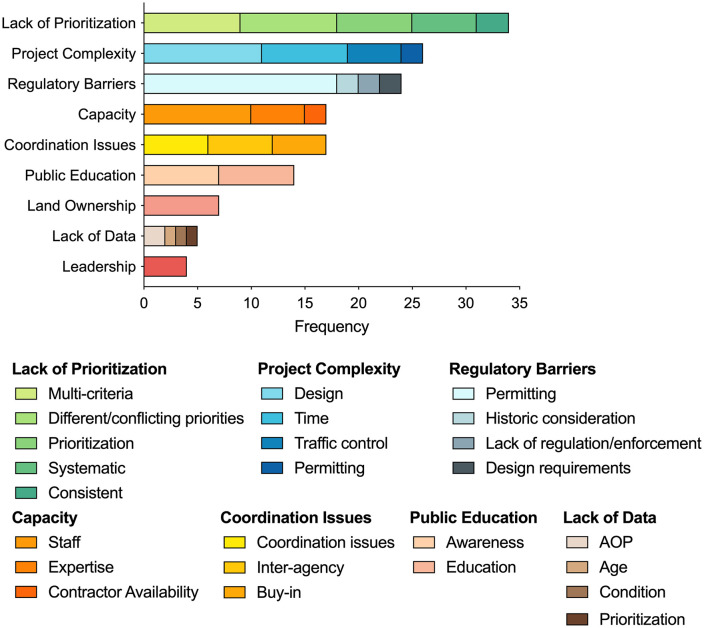
Frequency of non-financial challenges reported for road-stream crossing (RSC) management. The stacked bar chart shows the total number of coded mentions for each thematic category derived from inductive content analysis of responses to an open-ended survey question on non-financial barriers. Themes include Lack of Prioritization, Project Complexity, Regulatory Barriers, Capacity, Coordination Issues, Public Education, Land Ownership, Lack of Data, and Leadership. Colored segments within each bar represent specific codes (subcategories) within a theme, such as permitting, staff shortages, or contractor availability. Each segment corresponds to one or more coded statements from individual survey respondents; respondents could contribute multiple codes across themes.

The analysis of non-financial challenges in RSC management, presented in Fig 5, highlights four recurring themes: lack of prioritization, project complexity, regulatory barriers, and capacity constraints. These challenges affect stakeholders at different operational levels and impede efficient and sustainable RSC management.

Lack of prioritization emerged as the most frequently reported challenge, particularly among NHDES and NHDOT. These agencies grapple with a large number of assets at the state scale and face numerous competing demands under limited funding, often without a consistent or transparent process for prioritizing projects. The absence of standardized, transparent, and multi-criteria prioritization frameworks further complicates decision-making, leading to inconsistent management strategies across agencies. This finding underscores the need for prioritization tools and technical support that account for both shared and conflicting stakeholder values, as revealed in both stakeholder preference analyses.

Project complexity, including design requirements and traffic control, is also a major concern, particularly for MCCs, NHDOT, Municipalities, and Non-Profits. These stakeholders often struggle to balance technical standards (e.g., for flood resilience and habitat connectivity) with the practical need of maintaining traffic flow during construction. This challenge is especially pronounced in rural areas with limited alternate routes. Addressing this challenge requires streamlining design processes, such as promoting the use of standardized templates, modular components, or prefabricated structures. Additionally, integrating traffic control planning early in the design phase, such as through temporary bypasses or coordinated detours developed with local input, can help minimize disruption and delay.

Regulatory barriers, particularly permitting requirements, were commonly cited by Municipalities and NHDOT. Fragmented approval processes across state and federal agencies often introduce uncertainty and administrative burdens, discouraging proactive infrastructure upgrades. Establishing integrated permitting pathways can help reduce redundancies, harmonize regulatory expectations, and shorten review timelines. For smaller projects, expanding access to the Certified Culvert Maintainer (CCM) program could empower municipal staff to perform routine maintenance and minor upgrades without requiring extensive permitting processes. Developing detailed regulatory guidance, compliance manuals, and training workshops can further support municipalities in navigating complex regulations effectively.

Capacity constraints, particularly staffing shortages and limited technical expertise, pose a significant barrier to municipal-level RSC management. Small and rural municipalities often lack full-time engineers and planners and must rely on external consultants, which increases project costs and limits institutional continuity. Frequent staff turnover can further erode technical capacity and delay implementation. Expanding workforce training programs and technical certification initiatives, such as the CCM program, can equip municipal staff with the necessary expertise to oversee RSC projects more effectively. Additionally, regional coalitions and shared-resource networks can help distribute technical capacity across multiple municipalities, reducing dependency on external consultants while fostering cross-jurisdictional collaboration.

These findings point to clear opportunities for policy intervention to strengthen the equity and sustainability in RSC management. By addressing the identified non-financial challenges through transparent prioritization frameworks, streamlined regulatory processes, reduced permitting complexity, and robust capacity-building initiatives, agencies can accelerate infrastructure improvements while ensuring that projects are both equitable and ecologically sound.

## 4. Conclusions

This study examined stakeholder preferences, collaboration networks, and non-financial challenges in RSC management, identifying key opportunities and challenges for improving decision-making. FV emerged as the top priority with broad agreement among stakeholders, highlighting a common ground for collaboration, while socio-economic goals such as EJ, EI, and CSR were consistently rated as least important, also with relatively strong consensus. While RC, WCR, EQ, and SR received relatively high overall importance ratings, stakeholder disagreement on these goals was also notable, particularly for WCR and RC, where the most pronounced differences emerged between transportation-oriented and conservation-oriented stakeholders. Social network analysis revealed that central stakeholders, such as NHDOT and NHDES, function as hubs to connect stakeholders and facilitate collaborative management. However, their dominance can create potential power imbalances that could marginalize local and underrepresented groups. Additionally, our study identified lack of prioritization, project complexity, regulatory barriers, and capacity constraints as the four key non-financial barriers to effective RSC management in NH. These challenges underscore the need for systematic, inclusive, and multi-criteria approaches to enhance equitable and effective decision-making.

The findings of this study provide insights for enhancing RSC management at local, state, and regional levels. Given the divergence in stakeholder priorities, policymakers should engage stakeholders to co-develop integrated multi-criteria decision frameworks that balance transportation, ecological, and socio-economic objectives, ensuring that infrastructure planning is inclusive and transparent. Expanding collaborative initiatives such as the NHSCI to incorporate more diverse stakeholders, especially municipalities, conservation groups, and regional planning organizations, can help foster collaborative management while reducing polarization. Regulatory simplifications, such as streamlined permitting processes and clearer compliance guidance, can address procedural inefficiencies that disproportionately affect stakeholders. Additionally, investments in workforce capacity-building programs, technical training, and regional resource sharing can mitigate expertise shortages, particularly in smaller municipalities. These actions can improve the coordination, resilience, and equity of RSC management.

There are several limitations in our study that should be considered when interpreting the findings. First, while statistical methods controlled for variations in group size, some stakeholder groups had lower response rates (e.g., federal agencies and counties’ conservation districts), potentially leading to overrepresentation of certain priorities. Future research should ensure broader outreach to underrepresented groups to capture a more comprehensive range of perspectives. Second, although iterative refinement of the content analysis coding framework helped capture emergent themes, the qualitative content analysis may still oversimplify the interconnected nature of non-financial barriers, such as regulatory and project complexity. Future research could integrate participatory stakeholder workshops to refine these categorizations and better capture cross-cutting challenges. Third, although we discuss governance and policy strategies that stakeholders expect to improve procedural efficiency, this study does not directly quantify time, cost, or recovery-rate outcomes of RSC management. Evaluating such performance metrics lies outside the scope of this study. Finally, the study is specific to NH, where environmental policies, funding mechanisms, and governance structures shape stakeholder priorities. While the results offer valuable insights, their applicability to other regions should be assessed within the context of local regulatory and ecological conditions. Future work on expanding the scope to multiple states or conducting comparative analyses would provide a broader understanding of how governance structures shape RSC management challenges.

## Supporting information

S1 File Combined supplementary materials. This file contains the evaluation criteria within the overarching goals framework (Section S1), stakeholder engagement and survey distribution details, including Table S3 (Section S2), nodes of the decision-making network, including Table S2 (Section S3), results and discussions for evaluation criteria (Section S4), and the stakeholder-informed survey instrument (Section S5).
(PDF)

S2 File Treated and de-identified survey data. This file contains the anonymized dataset used for the analysis in CSV format.
(CSV)
