# Supplementary material for: Bridging priorities: Stakeholder preferences, networks, and barriers in road-stream crossing management
Source: PLoS One. 2026 Jan 5;21(1):e0339740. doi: 10.1371/journal.pone.0339740 (PMC12768365; doi:10.1371/journal.pone.0339740)
Supplement: S1 File — This file contains the evaluation criteria within the overarching goals framework (Section S1), stakeholder engagement and survey distribution details, including Table S3 (Section S2), nodes of the decision-making network, including Table S2 (Section S3), results and discussions for evaluation criteria (Section S4), and the stakeholder-informed survey instrument (Section S5). (PDF) [file pone.0339740.s001.pdf]

Supporting Information

Bridging Priorities: Stakeholder Preferences, Networks, and Barriers in Road-Stream Crossing Management

Contents

S1. Evaluation Criteria within the Overarching Goals Framework 2

S2. Stakeholder Engagement and Survey Distribution 5

S3. Nodes of Decision-Making Network 9

S4. Results and Discussions for Evaluation Criteria 12

S5. Stakeholder-Informed Survey 26

S1. Evaluation Criteria within the Overarching Goals Framework

Table S1 outlines the evaluation criteria associated with each overarching goal. These criteria represent measurable and quantifiable attributes that define progress toward each goal. They form the basis of the prioritization framework for road-stream crossing (RSC) management by enabling scoring for each goal. The criteria were co-developed in collaboration with our Technical Advisory Committee (TAC).

Table S1. Overarching goals and associated evaluation criteria for prioritizing road-stream crossing management

**Overarching goals (Strategic aspiration)**	**Evaluation criteria**	**Description of the evaluation criteria**
**Wildlife Conservation and Restoration**	Aquatic organism passage (AOP)	The ability of stream crossing to allow for AOP (i.e., passability). AOP is used to identify crossings that may be barriers to aquatic organisms and prohibit their movement through the stream.
	Watershed AOP	Considers the upstream and downstream organism movement blockages of a stream crossing. Upstream and downstream blockages determine whether managing the current stream crossing can be effective for restoring sea-run fish species. It views stream crossing management from a whole watershed scale.
	Terrestrial organism passage	The ability of the stream crossing to allow for terrestrial organisms (e.g., mammals, reptiles, etc.) to pass.
	Habitat quantity	The amount of upstream and downstream reconnected habitat when a stream crossing is replaced. Calculated from the replaced stream crossing to known natural or manmade barriers upstream and downstream.
	Habitat quality	The condition and ecological suitability of a stream environment, determined by factors such as water quality, stream flow, and the life cycle of aquatic organisms.
	Downstream and upstream blockage rate	The downstream and upstream blockage rate is calculated based on the lowest aquatic organism passability of the downstream and upstream barriers.
	Number of barriers	Number of upstream and/or downstream barriers
	Conservation status	Conservation status of lands within the upstream watershed, and up- and downstream buffer. It estimates the degree to which watershed and sub-watershed buffers were conserved as a way to estimate the potential for habitat to change over time.
	Wetland connectivity	The degree of wetland reconnection gained
	Special species presence and population	Whether a special species is present in the stream and its population (e.g., endangered or not), e.g., eastern brook trout
	Species diversity	The number of species present in the stream/watershed

**Overarching goals (Strategic aspiration)**	**Evaluation criteria**	**Description of the evaluation criteria**
	Stream order	A positive whole number used in geomorphology and hydrology to indicate the level of branching in a river system.
	Risk of wildlife collision	Risk of Wildlife-vehicle collisions within a certain distance from the stream crossing.
**Environmental Quality**	Geomorphic compatibility (GC)	Evaluates how well the stream crossing structure fits within the natural shape and form of the stream and whether it alters water and sediment transport. GC is derived from a model that uses the SADES survey data to rank the crossings from “fully compatible” to “fully incompatible”.
	Continuity of sediment, carbon, nutrients, large wood, and other transport constituents	Uninterrupted and stable transport and distribution of these elements through a riverine system, contributing to habitat formation, nutrient cycling, carbon sequestration, and overall ecosystem functioning and resilience.
	Erosion	The removal of sediment from around or beneath a stream crossing, as well as upstream or downstream due to the flow of water.
	Water use	Whether the water is used as a source for public drinking water supply or used for recharging groundwater aquifers
	Water quality impairment	Indicates if the stream crossing is on an impaired water body.
	Entrenchment Ratio	Calculated as the flood-prone width divided by the bankfull width. Entrenchment ratio is the vertical containment of a river as seen by the relationship between the channel (within the bankfull width) and the surrounding floodplain (within the flood prone width). The lower the ratio, the more entrenched a channel is.
**Road Criticality**	Annual average daily traffic (AADT)	Average daily traffic on a roadway link for all days of the week during a period of one year, expressed in VPD (vehicles per day)
	Road tier	Tier 1 – Interstates, Turnpikes, and Divided Highways<br>Tier 2 – Statewide Corridors<br>Tier 3 – Regional Transportation Corridors<br>Tier 4 – Local Connectors<br>Tier 5 – Local Roads<br>Tier 6 – Off Network
	Detour length	The total additional travel a through-bound vehicle would experience if a stream crossing fails.
	Location of important services	Stream crossing’s distance to the location of important services, such as hospitals, fire departments, police, etc.
	Public safety routes	Whether the stream crossing is on the routes of emergency evacuation or buses/school buses.

Overarching goals (Strategic aspiration)	Evaluation criteria	Description of the evaluation criteria
	Single road into a community	Whether the stream crossing is on a single road going in and out of the community.
**Economic Impact**	Long term economic benefits	Long term economic benefits to the local communities reflected through reduced flooding risk, better environmental quality, higher property value, better tourism routes, etc.
	Capital cost	The capital cost refers to the estimated total expense Incurred in acquiring, constructing, or upgrading a stream crossing.
	O&M cost	The O&M cost refers to the annual recurring cost for operating and maintaining the stream crossing.
	Repaving schedule	Indicates if the stream crossing on a road scheduled to be repaved in the future, such as the next 5, 10, or 15 years.
	Funding availability and accessibility	The ease and availability of financial resources or capital for individuals, organizations, or projects. It encompasses the extent to which funding options and opportunities are accessible, Inclusive, and readily obtainable by diverse individuals or groups.
**Flood Vulnerability**	Hydraulic capacity	Predicts how a stream crossing will transport flows during storm events. This information is helpful to make mindful decisions on flood probability prediction and identifying the most vulnerable infrastructure.
	Geomorphic Compatibility (GC)	The Geomorphic Compatibility (GC) score evaluates how well the stream crossing structure fits within the natural shape and form of the stream and whether it alters water and sediment transport. This is completed to predict the long-term compatibility of a stream crossing with river channel form. The GC score is derived from a model that uses the survey data to rank the crossings from “fully compatible” to “fully Incompatible”.
	Flood days per year	Estimated number of days where water overtops the road surface during an average years' worth of rainfall.
	Documented history of flooding	Whether there has been any documented history of the culvert being washed out/flooded.
	Climate resiliency	Changes in the occurrence, magnitude, and characteristics of flooding events due to climate change, and how climate change may influence evacuation during a flooding event.
**Structural Risk**	Structural condition	The physical state of a stream crossing encompasses its material integrity, overall stability, and ability to safely convey stream flows without compromising its structure or the infrastructure it supports.
	Age of the stream crossing	Age of the stream crossing.
	Material	Material of the stream crossing structure.
	Size and depth	Attributes of a stream crossing (depth of cover and cross-sectional area) that may influence the magnitude of a stream crossing replacement project as well as the impact of failure to public safety.
	Stream fit	How well the stream crossing structure fits within the natural shape and form of the stream channel (percent bankfull width) and

Overarching goals (Strategic aspiration)	Evaluation criteria	Description of the evaluation criteria
		whether flow (hydraulic capacity) could negatively impact the structure, itself.
**Community Support and Readiness**	Community preparedness	Whether the stream crossing is referenced in Master Plans, flood hazard database, municipal planning documents such as Hazard, Master, or Capital Improvement Plans, Regional planning documents such as Transportation, Corridor, or Regional Plans.
	State and federal support	Stream crossings or areas that contain stream crossings that have been identified by state and/or federal agencies as a priority.
	Funding availability and accessibility	The ease and availability of financial resources or capital for individuals, organizations, or projects. It encompasses the extent to which funding options and opportunities are accessible, Inclusive, and readily obtainable by diverse individuals or groups.
**Environmental Justice**	Distributional equity	Fairness and equality in the distribution of resources for stream crossing replacement, benefits gained from stream crossing replacement, or management burdens among individuals or groups within a society.
	Procedural equity	Ensuring that the stream crossing replacement decision-making and implementation processes employed are Inclusive, unbiased, consistent, and provide individuals with a sense of fairness and legitimacy.

S2. Stakeholder Engagement and Survey Distribution

During the outreach phase, we conducted a series of in-person and virtual meetings with key stakeholder organizations to maximize participation and foster understanding of the overarching goals and associated evaluation criteria. These engagements provided a forum for stakeholders to voice their concerns and helped build a shared understanding of how the goals and criteria could be integrated into future decision-making. Importantly, these conversations laid the groundwork for more coordinated and equitable management practices by highlighting the practical benefits of applying the framework developed through the subsequent survey. Below is the list of stakeholders we engaged with during the outreach phase.

1. New Hampshire Department of Agriculture, Markets, and Food
2. Rockingham Regional Planning Commission
3. Lakes Region Planning Commission
4. Upper Valley Lake Sunapee Regional Planning Commission
5. Southwest Regional Planning Commission
6. Nashua Regional Planning Commission
7. Southern New Hampshire Planning Commission
8. Strafford Regional Planning Commission

9. Lake Management Advisory Committee
10. Cheshire County Conservation District
11. Grafton County Conservation District
12. University of New Hampshire Technology Transfer Center

Following this outreach, we distributed a survey via email to representatives from a broader set of stakeholders, including state agencies, conservation districts, regional planning commissions, municipalities, non-profits, academic institutions, and private sector consultants (Table S2).

Table S2. Categorized list of stakeholder organizations engaged through outreach and survey distribution phases, including government agencies, planning commissions, conservation districts, municipalities, academic institutions, non-profits, and private-sector consultants.

Category	Stakeholder Organization
**Academic Institutions**	Keene State College
	Plymouth State University
	University of New Hampshire Stormwater Center
	University of New Hampshire Technology Transfer Center
**Conservation Districts**	Belknap County Conservation District
	Carroll County Conservation District
	Cheshire County Conservation District
	Coos County Conservation District
	Grafton County Conservation District
	Hillsborough County Conservation District
	Merrimack County Conservation District
	Rockingham County Conservation District
	Strafford County Conservation District
	Sullivan County Conservation District
**Federal Agencies**	
	U.S. Forest Service
	U.S. Department of Agriculture
	U.S. Fish and Wildlife Service
	U.S. Environmental Protection Agency
	National Oceanic and Atmospheric Administration
	Natural Resources Conservation Service
	Federal Highway Administration
	Federal Emergency Management Agency
	United States Army Corps of Engineers
	United States Geological Survey
**Municipalities**	City of Dover
	City of Portsmouth
	City of Rochester
	Town of Alstead
	Town of Durham

	Town of Exeter
	Town of Hampton
	Town of Marlborough
	Town of Plymouth
	Town of Warner
	Town of Winchester
**Private Sector Consultants and Engineering Firms**	Aceti Associates
	Acorn Engineering, Inc.
	Aldrich & Elliott, P.C.
	Allen & Major Associates, Inc.
	Arcadis
	Aries Engineering, LLC
	Bedford Design Consultants
	Beta Group, Inc.
	CMA Engineers, Inc.
	Civilworks New England
	Comprehensive Environmental, Inc.
	DK Water Resource Consulting, LLC
	DuBois & King, Inc.
	E.J. Prescott, Inc.
	Ecological Instincts
	FB Environmental
	Field Geology Services
	Fitzgerald Environmental Associates, LLC
	Fuss & O'Neill
	GHD
	GM2 Associates, Inc.
	Gale Associates, Inc.
	GeoInsight, Inc.
	Geosyntec Consultants, Inc.
	Golder Associates
	Gomez & Sullivan Engineers
	Gorrill Palmer Consulting Engineers
	Gove Environmental Services
	HEB Engineers, Inc.
	Hazen and Sawyer, Inc.
	Headwaters Consulting, LLC
	Holden Engineering & Surveying, Inc.
	Horizons Engineering, Inc.
	Horsley Witten Group
	Hoyle, Tanner & Associates, Inc.
	HydroAnalysis LLC
	Ibis Consulting Group

	Inter-Fluve, Inc.
	Jake Dawson's Excavation & Utility Services, LLC
	John Turner Consulting, Inc.
	Jones & Beach Engineers, Inc.
	KV Partners LLC
	Kleinfelder & SEA Consultants
	Loureiro Engineering Associates, Inc.
	Miller Engineering & Testing, Inc.
	NH Springs Environmental Consulting LLC
	Nobis Engineering, Inc.
	Northeast Conservation Services, LLC
	Northeast Earth Mechanics, Inc.
	Northeast Wetland Restoration
	Northpoint Engineering, LLC
	Oak Point Associates
	Pathways Consulting, LLC
	Pike Industries, Inc.
	R.S. Audley, Inc.
	S.W. Cole Engineering, Inc.
	SLR Consulting
	SVE Associates
	Stantec Consulting Services, Inc.
	Stephens Associates Consulting Engineers, LLC
	Stone Environmental, Inc.
	Stoney Ridge Environmental LLC
	Streamworks, PLLC
	T.F. Bernier, Inc.
	TFMoran, Inc.
	Tata & Howard, Inc.
	The Dufresne Group, Inc.
	The H.L. Turner Group Inc.
	Tighe & Bond, Inc.
	Truslow Resource Consulting, LLC
	Underwood Engineers, Inc.
	VHB (Vanasse Hangen Brustlin, Inc.)
	Waterstone Engineering, PLLC
	Weston & Sampson Engineers, Inc.
	Wilcox & Barton, Inc.
	Woodard & Curran, Inc.
	Wright-Pierce, Inc.
	Naturesource communications
**Regional Planning Commissions**	Central New Hampshire Regional Planning Commission
	Lakes Region Planning Commission

	Nashua Regional Planning Commission
	North Country Council
	Rockingham Regional Planning Commission
	Southern New Hampshire Planning Commission
	Southwest Regional Planning Commission
	Strafford Regional Planning Commission
	Upper Valley Lake Sunapee Regional Planning Commission
**State Agencies**	New Hampshire Department of Agriculture, Markets, and Food
	New Hampshire Department of Environmental Services
	New Hampshire Department of Transportation
	New Hampshire Fish and Game Department
	New Hampshire Homeland Security and Emergency Management
**Watershed and Environmental Organizations**	Acton Wakefield Watersheds Alliance
	American Rivers, Inc.
	Big Pea Porridge Pond Preservation Association
	Connecticut River Conservancy
	Connecticut River Valley Resource Commission
	Green Mountain Conservation Group
	Lake Management Advisory Committee
	Merrimack River Watershed Council
	Messer Pond Protective Association
	New Hampshire Association of Conservation Commissions
	New Hampshire Rivers Council
	Newfound Lake Region Association
	Pine River Pond Association
	Piscataqua Region Estuaries Partnership
	River Advisory Committee
	Saco Headwaters Alliance
	The Nature Conservancy
	Trout Unlimited
	Upper Merrimack Watershed Association

S3. Nodes of Decision-Making Network

To identify central actors who can support more coordinated RSC management, we asked survey respondents to list the organizations with which they have collaborated. Based on these responses, we conducted a social network analysis, where each stakeholder organization was represented as a node, and a connection (edge) was drawn between nodes when a collaboration was documented in the survey. Table S3 summarizes the nodes included in the stakeholder network. For each node, the table provides the official name of the stakeholder, as well as example responses given by survey participants that were grouped under that node.

Table S3. Stakeholder nodes identified in the decision-making network, based on survey responses indicating collaboration.

Network Node	Stakeholder	Example Collaboration Input
NHFG	New Hampshire Department of Fish and Game	"Fish and Game"
		"NHFGD"
		"F&G"
NHDES	New Hampshire Department of Environmental Services	"DES"
		"ARM Fund Site Selection Committee"
		"DES Wetlands Bureau"
NHDOT	New Hampshire Department of Transportation	"DOT"
		"Bureau of Bridge design, bridge maintenance, and bureau of AMPS"
		"NHDOT Bureau of Environment"
NHSCI	New Hampshire Stream Crossing Initiative	"NHSCI"
NHDA	New Hampshire Department of Agriculture	"NHDAMF" (NH Department of Agriculture, Markets and Food)
NHDBEA	New Hampshire Department of Business and Economic Affairs	"Office of Planning and Development"
NHDNCR	New Hampshire Department of Natural & Cultural Resources	"NH Division of Historical Resources"
		"DNCR"
NHNHB	New Hampshire Natural Heritage Bureau	"NHNHB"
NHHSEM	New Hampshire Homeland Security and Emergency Management	"NH HSEM"
NHDOE	New Hampshire Department of Energy	"NHDOE"
NHCAW	New Hampshire Coastal Adaptation Workgroup	"CAW"
USFS	U.S. Forest Service	"USFS"
		"US Forest Service"
USDA	U.S. Department of Agriculture	"USDA"
USFWS	U.S. Fish and Wildlife Service	"FWS"
USEPA	U.S. Environmental Protection Agency	"EPA"
NOAA	National Oceanic and Atmospheric Administration	"NOAA"
NRCS	Natural Resources Conservation Service	"NRCS"

FHWA	Federal Highway Administration	"FHWA"
FEMA	Federal Emergency Management Agency	"FEMA"
USACE	United States Army Corps of Engineers	"USACE"
		"ACOE"
USGS	United States Geological Survey	"Geological Survey"
Municipalities	Municipalities	"Work with local highway department for stream crossings on municipal highways"
		"local road agents"
		"Town staff"
		"Town's consulting engineers"
		"town officials/road agents"
		"towns"
MCCs	Municipal Conservation Commissions	"conservation commissions"
		"Town Conservation Committees"
CCDs	County Conservation Districts	"County Conservation Districts"
		"Grafton County Conservation District"
		"Cheshire County Conservation District"
Universities	University of New Hampshire	"NHT2 (UNH Technology Transfer)"
		"UNH"
	Plymouth State University	"PSU Environmental program"
		"Plymouth State University"
Non-Profit Organizations	Watershed and/or Lake Associations	"watershed/lake associations"
	The Nature Conservancy	"The Nature Conservancy"
		"TNC"
	Trout Unlimited	"Trout Unlimited"
		"TU"
	Non-Governmental Organizations	"NGOs"
	Conservation Organizations	"Conservation organizations"
		"Nearly every conservation organization in NH"
		"Green Mountain Conservation Group"
		"NAACC" (*North Atlantic Aquatic Connectivity Collaborative*)
		"Saco Headwaters Alliance"
		"Wildlife agencies"
		"local watershed groups"
		"environmental non-profits"

		"Connecticut River Conservancy"
		"Pea Porridge Pond watershed Preservation Association"
		"Pine River Pond Lake Assocition (PRP Association) "
		"Mirror Lake Protective Association"
		"Baker River Watershed Association"
		"NH Association of Conservation Commissions"
		"Wildlife and Transportation Workgroup"
		"White Mountain National Forest Service"
RPCs	Regional Planning Commissions	"regional planning commissions"
		"North Country Council"
		"RPC"
		"Nashua Regional Planning Commission"
		"Rockingham Planning Commission"
LACs	Local Advisory Committees	"Contoccook and north branch rivers local advisory committee (lac)"
		"Pemi. River Local Advisory Committee"
		"local river advisory committee member"
Consultants	Engineering Consulting Firms	"Engineering Consultants"
		"consultants"
		"Hydraulic modelers from other consulting firms"
Public		"public"

S4. Results and Discussions for Evaluation Criteria

We applied the relative importance index (RII) method to evaluate the perceived priority of criteria associated with each overarching goal (Table S4). The results reflect stakeholder values that can guide future RSC management.

Under Wildlife Conservation and Restoration, aquatic organism passage (AOP) received the highest score (RII = 0.78), underscoring the current deficiency of the RSCs' abilities in AOP. This result supports prioritizing crossings with impaired AOP and integrating passage considerations into new designs. Special species presence (RII = 0.73) and wetland connectivity (RII = 0.70) were also highly ranked, reinforcing the importance of habitat-specific metrics in ecological planning.

For Environmental Quality, erosion (RII = 0.80) and geomorphic compatibility (RII = 0.77) were the most important criteria, emphasizing the need to address bank stability and preserve the natural stream shape in design standards. These results point to opportunities for incorporating geomorphic assessments into permitting and funding guidelines.

In the Road Criticality category, the highest-ranked criterion was whether a crossing serves as a single road into a community (RII = 0.70), highlighting a key vulnerability in rural areas. This underscores the need for improved spatial data and planning to ensure reliable access for isolated communities.

Under Flood Vulnerability, hydraulic capacity (RII = 0.86) and documented history of flooding (RII = 0.80) received high importance, reflecting concern over climate-exacerbated flood risk. These findings support the incorporation of previous flood records and hydraulic capacity measures into flood risk evaluations.

Structural condition (RII = 0.83) ranked first for the Structural Risk goal, affirming the importance of regular maintenance and inspection. Size and depth of cover (RII = 0.77) reflect the magnitude of failure when a crossing fails. The larger and deeper the crossing, the more challenging and expensive its management becomes. These criteria should remain central to asset management protocols.

In Economic Impact and Community Support and Readiness, funding availability and accessibility received high importance (RII = 0.81), indicating that financial feasibility is a critical enabler across these goals. In contrast, criteria associated with Environmental Justice received moderate scores (procedural equity RII = 0.58; distributional equity RII = 0.54), suggesting a need for clearer frameworks and guidance to embed equity considerations in project selection.

Table S4. Relative importance index (RII) for evaluation criteria associated with each goal.

Overarching goals (Strategic aspiration)	Evaluation criteria	RII
**Wildlife Conservation and Restoration**	Aquatic organism passage (AOP)	0.78
	Watershed AOP	0.71
	Terrestrial organism passage	0.67
	Habitat quantity	0.68
	Habitat quality	0.69
	Downstream and upstream blockage rate	0.68
	Number of barriers	0.63
	Conservation status	0.62
	Wetland connectivity	0.70
	Special species presence and population	0.73
	Species diversity	0.60

Overarching goals (Strategic aspiration)	Evaluation criteria	RII
	Stream order	0.53
	Risk of wildlife collision	0.57
**Environmental Quality**	Geomorphic compatibility (GC)	0.77
	Continuity of sediment, carbon, nutrients, large wood, and other transport constituents	0.7
	Erosion	0.8
	Water use	0.6
	Water quality impairment	0.66
	Entrenchment Ratio	0.61
**Road Criticality**	Annual average daily traffic (AADT)	0.59
	Road tier	0.54
	Detour length	0.5
	Location of important services	0.58
	Public safety routes	0.64
	Single road into a community	0.7
**Economic Impact**	Long term economic benefits	0.6
	Capital cost	0.68
	O&M cost	0.6
	Repaving schedule	0.41
	Funding availability and accessibility	0.81
**Flood Vulnerability**	Hydraulic capacity	0.86
	Geomorphic Compatibility (GC)	0.78
	Flood days per year	0.74
	Documented history of flooding	0.80
	Climate resiliency	0.78
**Structural Risk**	Structural condition	0.83
	Age of the stream crossing	0.53
	Material	0.56
	Size and depth	0.77
	Stream fit	0.75
**Community Support and Readiness**	Community preparedness	0.63
	State and federal support	0.74
	Funding availability and accessibility	0.81
**Environmental Justice**	Distributional equity	0.54
	Procedural equity	0.58

Figures S1–S8 show the response distributions and Kruskal–Wallis (KW) test results for evaluation criteria across the eight overarching goals. These results highlight where statistically significant differences exist in stakeholders’ perceptions of criterion importance. For criteria with significant KW results, Dwass-Steel-Critchlow-Fligner (DSCF) post-hoc pairwise comparisons

(Figures S9–S14) identified specific stakeholder groups in disagreement, indicating where future reconciliation is needed.

Several high-priority criteria showed significant differences, including aquatic organism passage, habitat quantity and quality, annual average daily traffic (AADT), detour length, and public safety routes. These differences highlight diverging ecological and infrastructure priorities, particularly between regulatory and implementation-focused groups. Notably, flood vulnerability criteria showed no significant disagreement, suggesting a shared concern that may serve as a policy entry point for broader collaboration.

The most frequent pairwise differences (DSCF p-value < 0.05) occurred between Regional Planning Commissions (RPCs) and NH Fish & Game (NHFG) (17), NHDOT and NHFG (15), and Municipalities and NHFG (13), reflecting key tensions between conservation and infrastructure stakeholders. Addressing these differences is essential for advancing coordinated, multi-benefit RSC management frameworks.

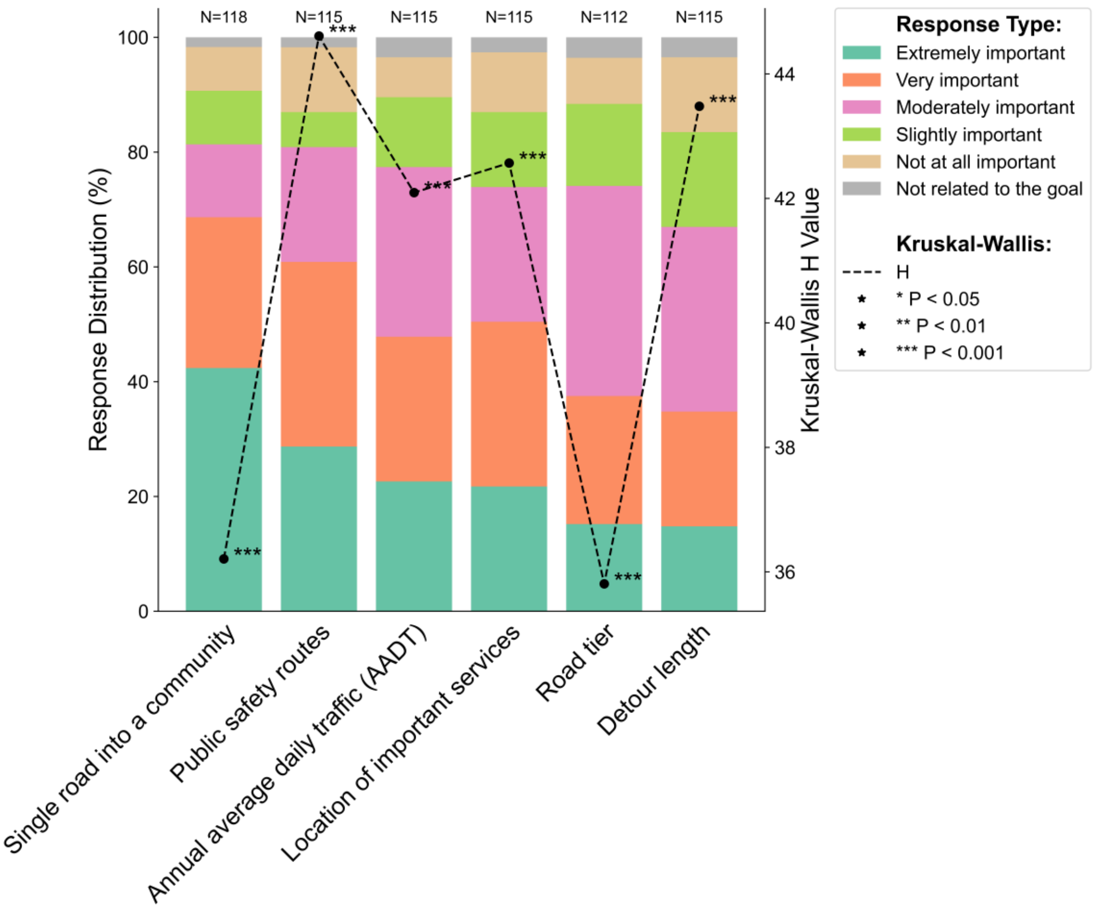

Figure S.1. The response distributions and the results of the Kruskal-Wallis statistical analysis for the evaluation criteria of the ***road criticality*** *goal for road-stream crossing prioritization. On the horizontal axis, criteria are listed; each bar shows the percentage of responses in six categories, from "Extremely*

important" down to "Not at all important," plus "Not related to the goal," with the total number of responses (N) indicated above each bar. Superimposed black dots and a dashed connecting line denote the Kruskal–Wallis H statistic for each criterion. Statistical significance is denoted by asterisks (for p < 0.05, ** for p < 0.01, *** for p < 0.001).*

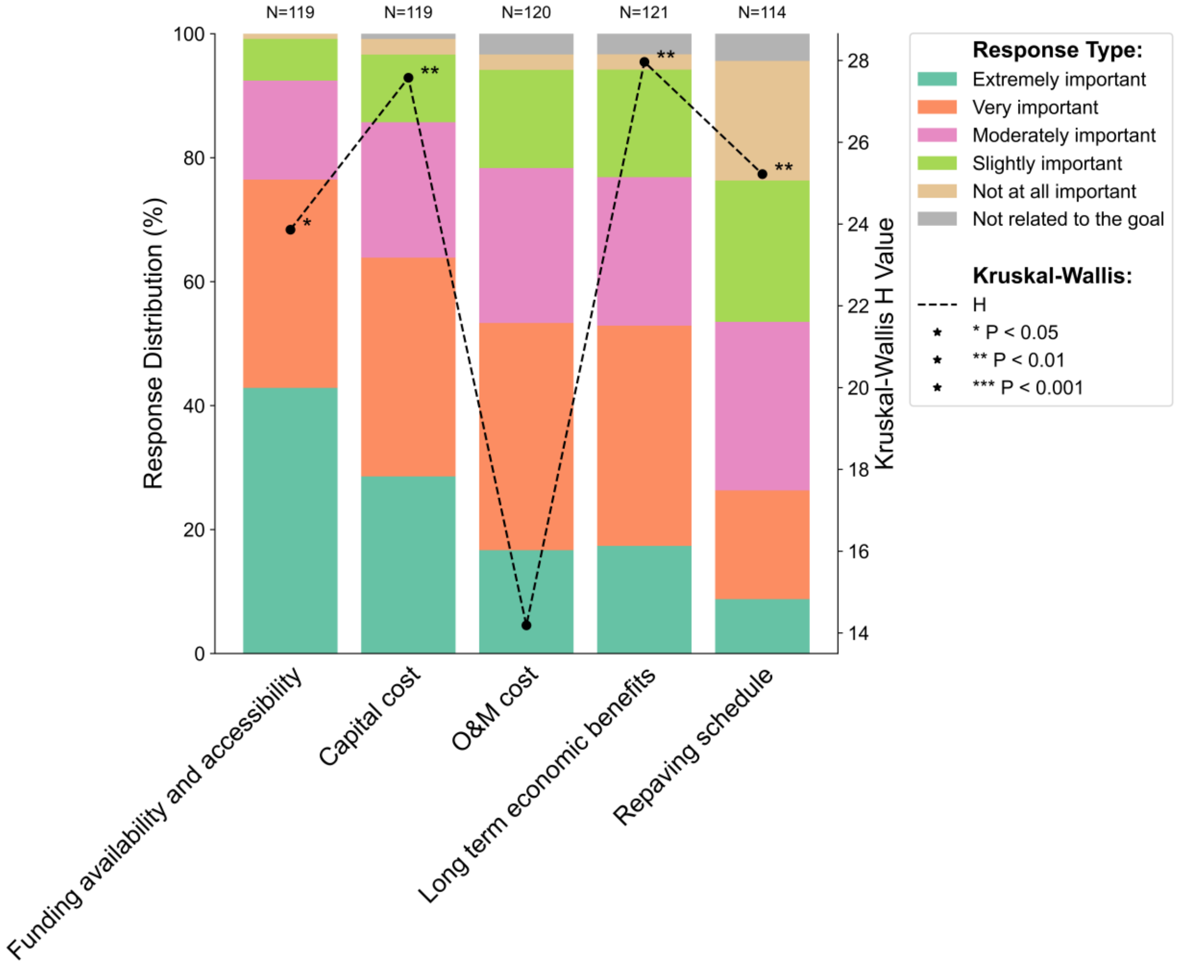

Figure S.2. The response distributions and the results of the Kruskal-Wallis statistical analysis for the evaluation criteria of the ***economic impact*** *goal for road-stream crossing prioritization. On the horizontal axis, criteria are listed; each bar shows the percentage of responses in six categories, from "Extremely important" down to "Not at all important," plus "Not related to the goal," with the total number of responses (N) indicated above each bar. Superimposed black dots and a dashed connecting line denote the Kruskal–Wallis H statistic for each criterion. Statistical significance is denoted by asterisks (* for p < 0.05, ** for p < 0.01, *** for p < 0.001).*

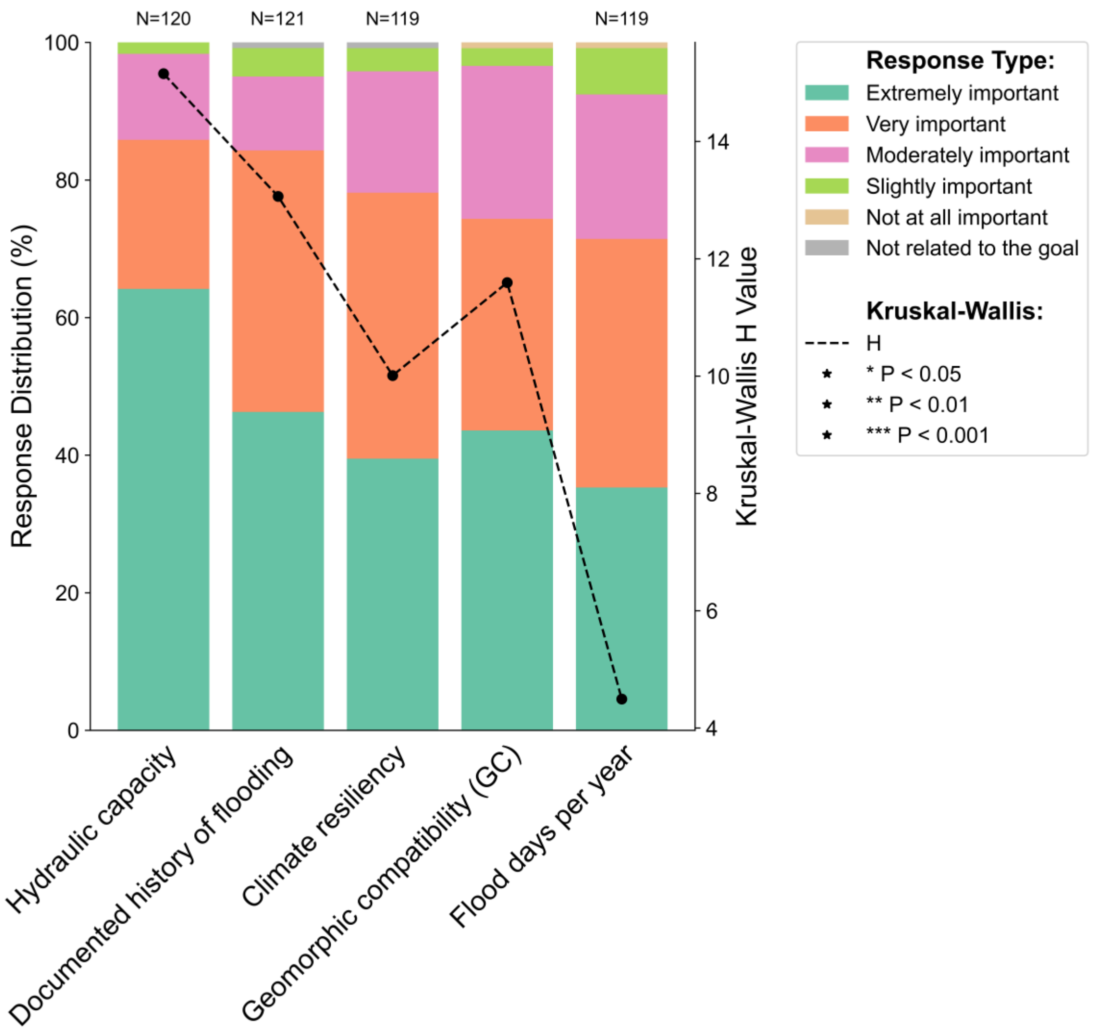

Figure S.3. The response distributions and the results of the Kruskal-Wallis statistical analysis for the evaluation criteria of the ***flood vulnerability*** *goal for road-stream crossing prioritization. On the horizontal axis, criteria are listed; each bar shows the percentage of responses in six categories, from "Extremely important" down to "Not at all important," plus "Not related to the goal," with the total number of responses (N) indicated above each bar. Superimposed black dots and a dashed connecting line denote the Kruskal–Wallis H statistic for each criterion. Statistical significance is denoted by asterisks (* for p < 0.05, ** for p < 0.01, *** for p < 0.001).*

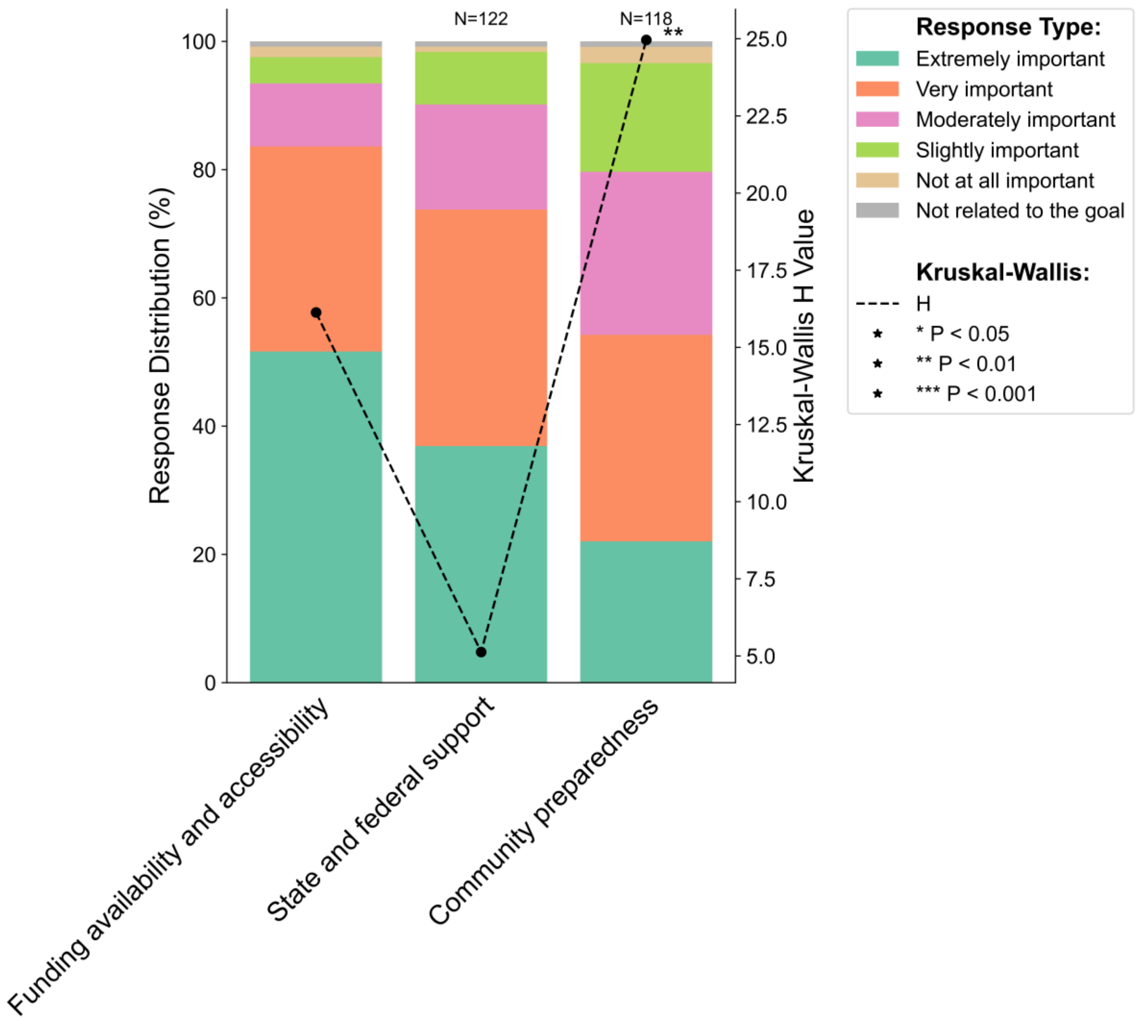

Figure S.4. The response distributions and the results of the Kruskal-Wallis statistical analysis for the evaluation criteria of the ***community support and readiness*** *goal for road-stream crossing prioritization. On the horizontal axis, criteria are listed; each bar shows the percentage of responses in six categories, from "Extremely important" down to "Not at all important," plus "Not related to the goal," with the total number of responses (N) indicated above each bar. Superimposed black dots and a dashed connecting line denote the Kruskal–Wallis H statistic for each criterion. Statistical significance is denoted by asterisks (* for p < 0.05, ** for p < 0.01, *** for p < 0.001).*

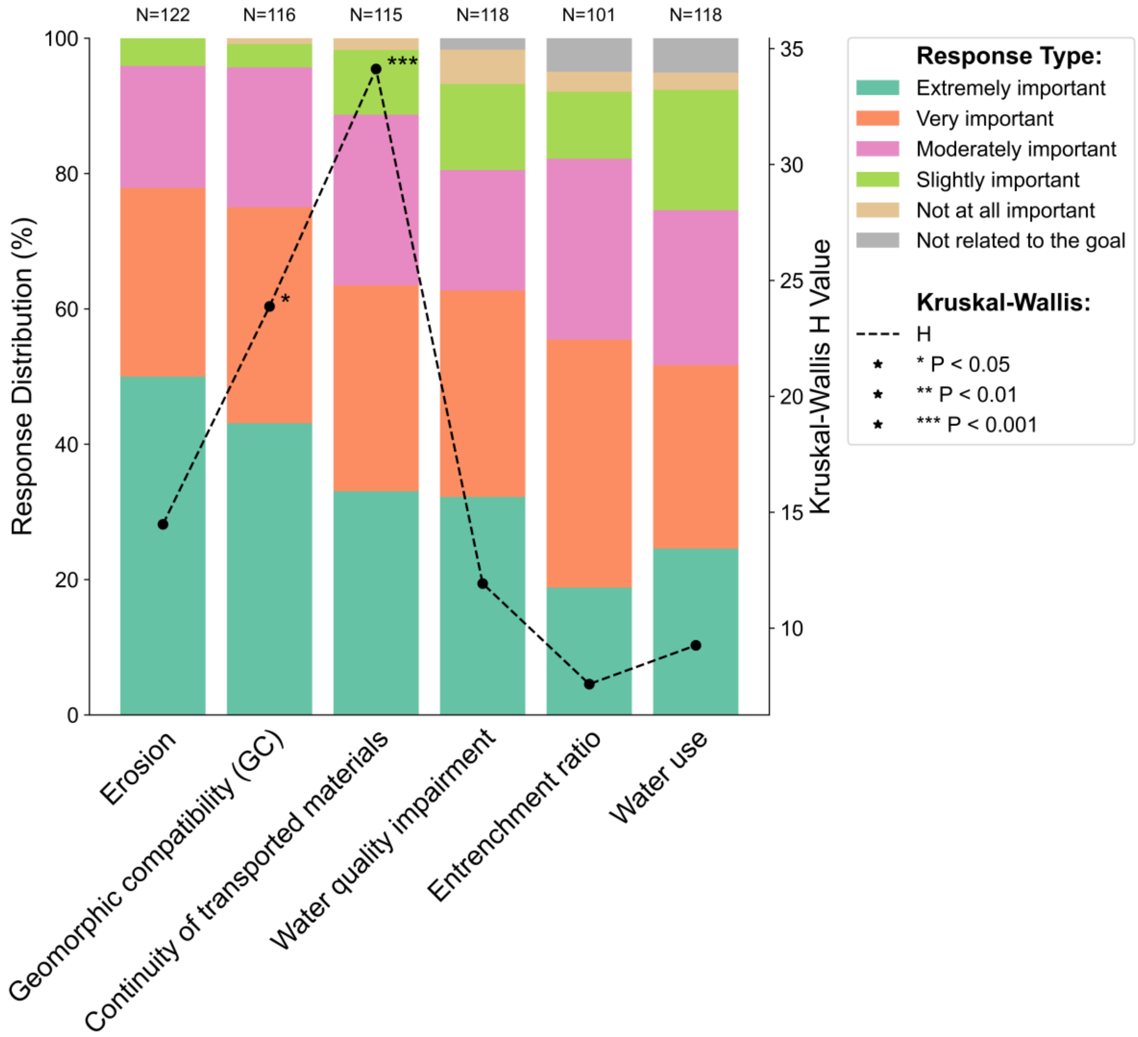

Figure S.5. The response distributions and the results of the Kruskal-Wallis statistical analysis for the evaluation criteria of the ***environmental quality*** *goal for road-stream crossing prioritization. On the horizontal axis, criteria are listed; each bar shows the percentage of responses in six categories, from "Extremely important" down to "Not at all important," plus "Not related to the goal," with the total number of responses (N) indicated above each bar. Superimposed black dots and a dashed connecting line denote the Kruskal–Wallis H statistic for each criterion. Statistical significance is denoted by asterisks (* for $p < 0.05$, ** for $p < 0.01$, *** for $p < 0.001$).*

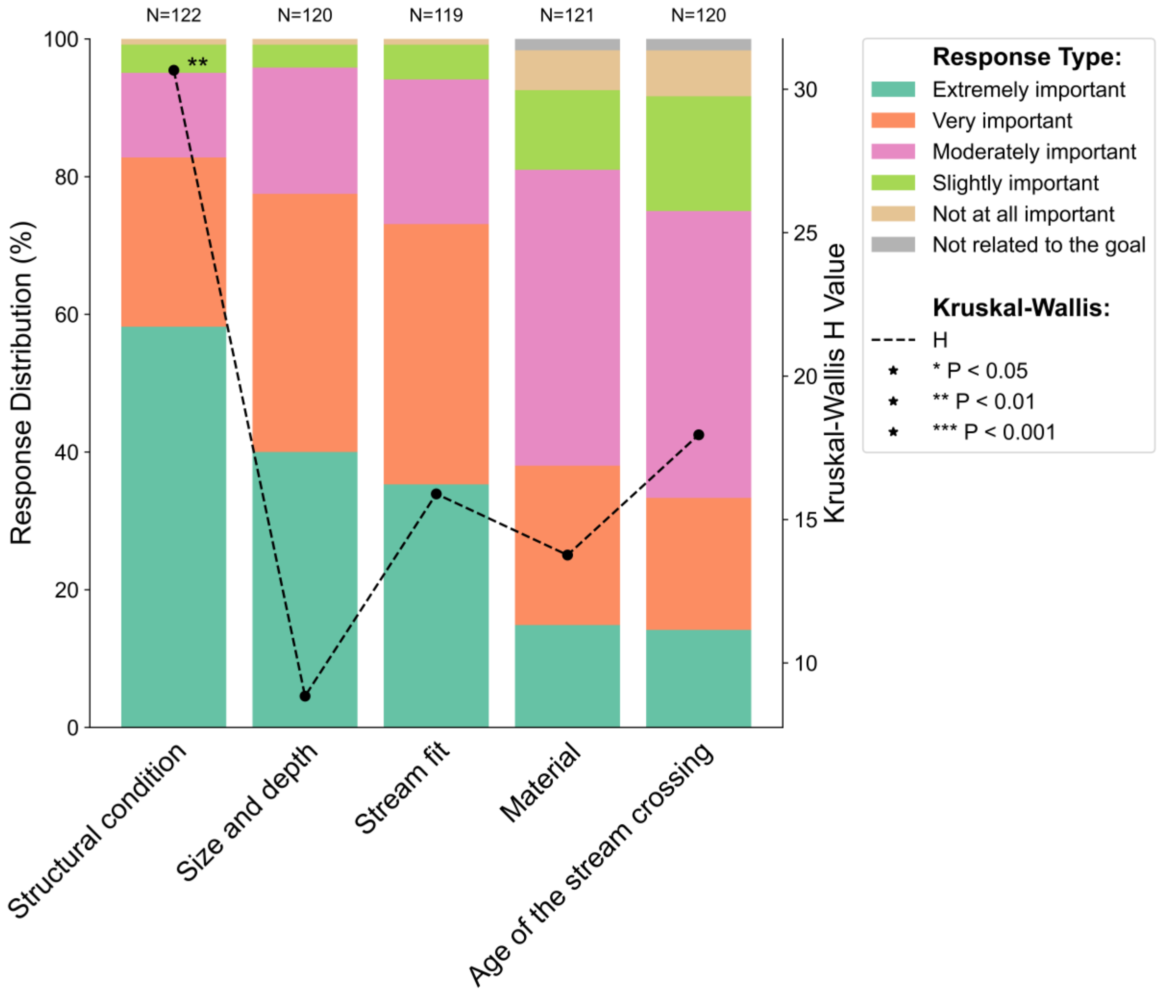

*Figure S.6. The response distributions and the results of the Kruskal-Wallis statistical analysis for the evaluation criteria of the **structural risk** goal for road-stream crossing prioritization. On the horizontal axis, criteria are listed; each bar shows the percentage of responses in six categories, from "Extremely important" down to "Not at all important," plus "Not related to the goal," with the total number of responses (N) indicated above each bar. Superimposed black dots and a dashed connecting line denote the Kruskal–Wallis H statistic for each criterion. Statistical significance is denoted by asterisks (* for p < 0.05, ** for p < 0.01, *** for p < 0.001).*

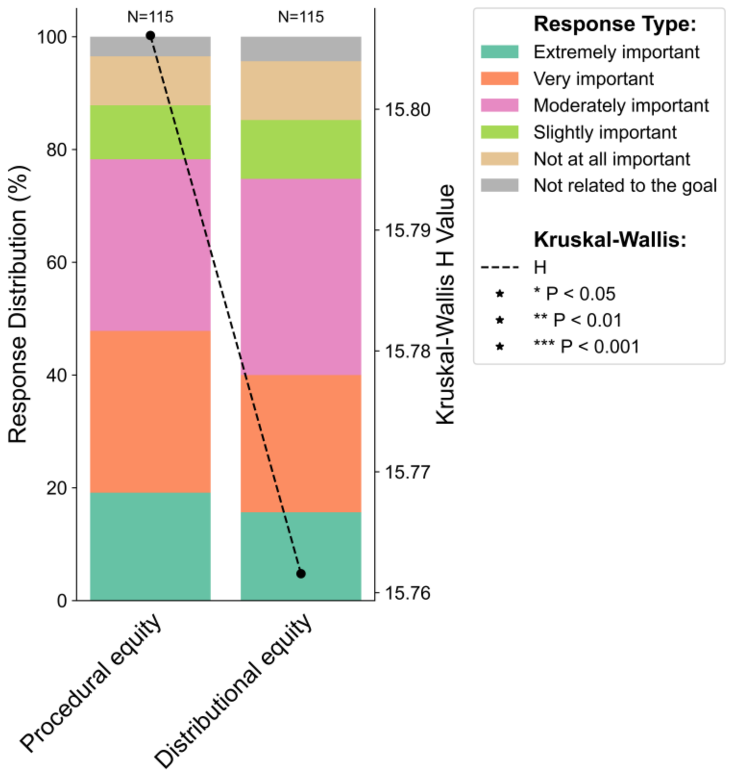

Figure S.7. The response distributions and the results of the Kruskal-Wallis statistical analysis for the evaluation criteria of the ***environmental justice*** *goal for road-stream crossing prioritization. On the horizontal axis, criteria are listed; each bar shows the percentage of responses in six categories, from "Extremely important" down to "Not at all important," plus "Not related to the goal," with the total number of responses (N) indicated above each bar. Superimposed black dots and a dashed connecting line denote the Kruskal–Wallis H statistic for each criterion. Statistical significance is denoted by asterisks (* for $p < 0.05$, ** for $p < 0.01$, *** for $p < 0.001$).*

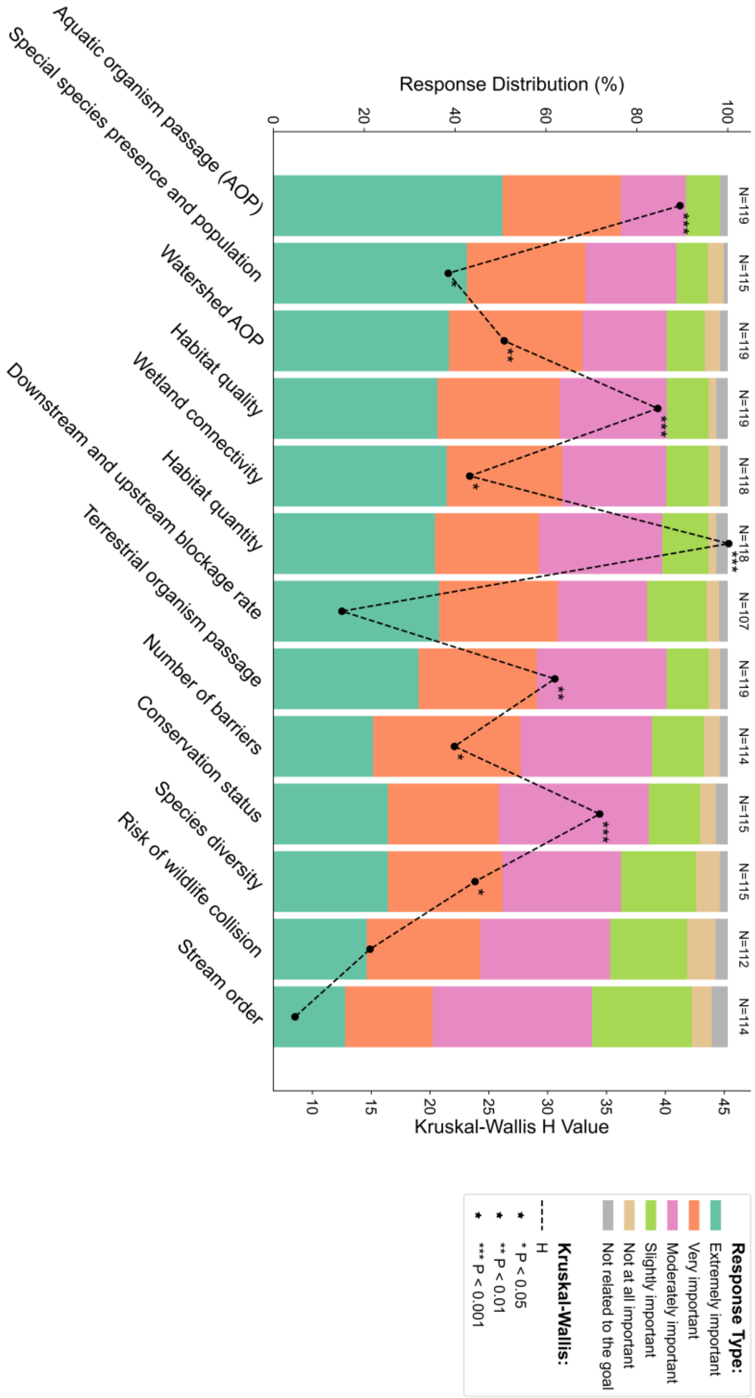

Figure S.8. The response distributions and the results of the Kruskal-Wallis statistical analysis for the evaluation criteria of the ***wildlife conservation and restoration*** *goal for road-stream crossing prioritization. On the horizontal axis, criteria are listed; each bar shows the percentage of responses in six categories, from "Extremely important" down to "Not at all important," plus "Not related to the goal," with the total number of responses (N) indicated above each bar. Superimposed black dots and a dashed connecting line denote the Kruskal–Wallis H statistic for each criterion. Statistical significance is denoted by asterisks (* for p < 0.05, ** for p < 0.01, *** for p < 0.001).*

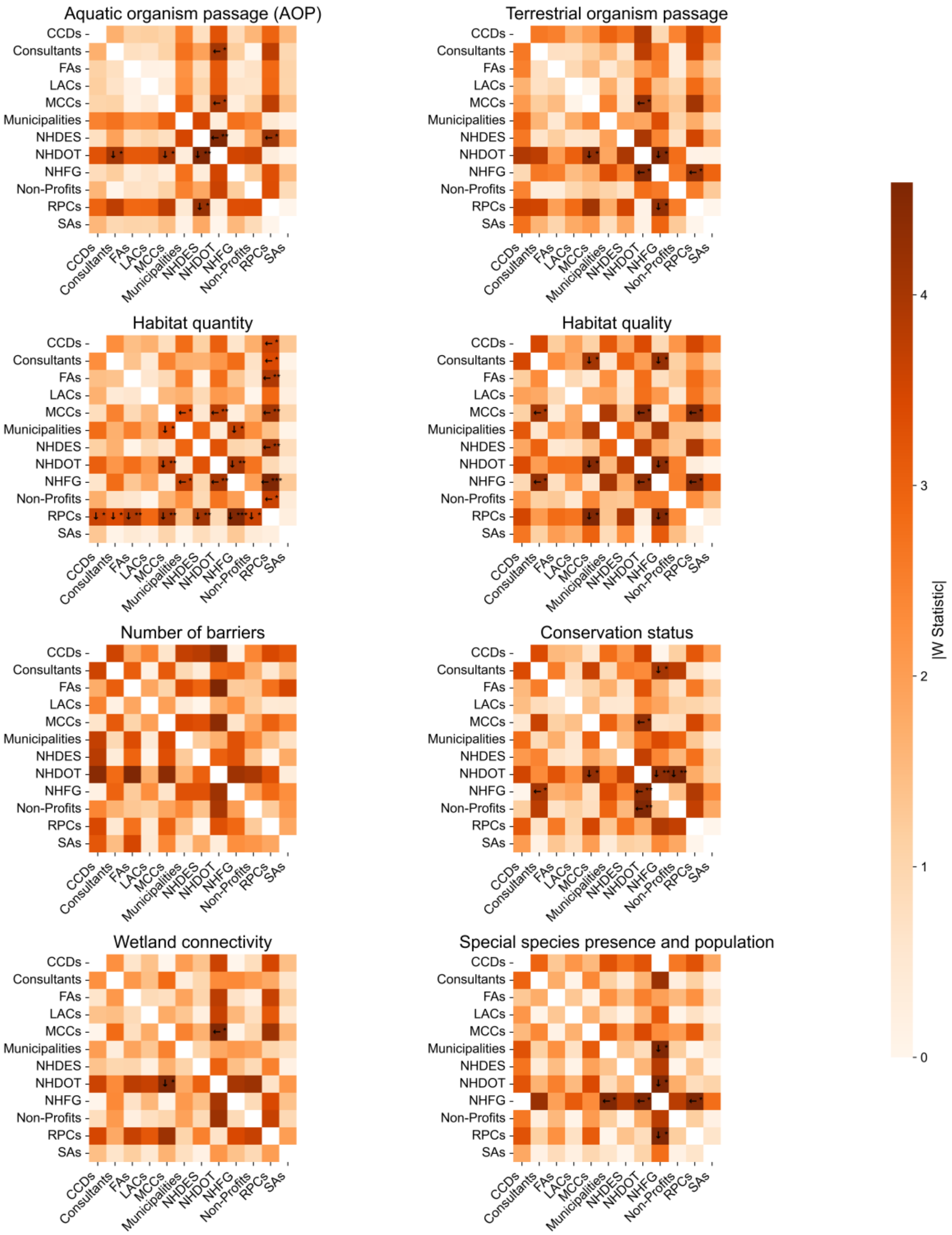

Figure S.9. Pairwise stakeholder differences in road-stream crossing (RSC) prioritization, based on the Dwass-Steel-Critchlow-Fligner (DSCF) post hoc test results for evaluation criteria of the ***wildlife conservation and restoration*** *with significant Kruskal-Wallis results. The matrix displays the absolute value of the test statistic (|W|), where darker shades represent greater differences in prioritization between stakeholder groups. Arrows indicate the direction of higher ratings, identifying which group assigned greater importance to the goal. Statistical significance is marked by asterisks (* p < 0.05, ** p < 0.01, *** p < 0.001).*

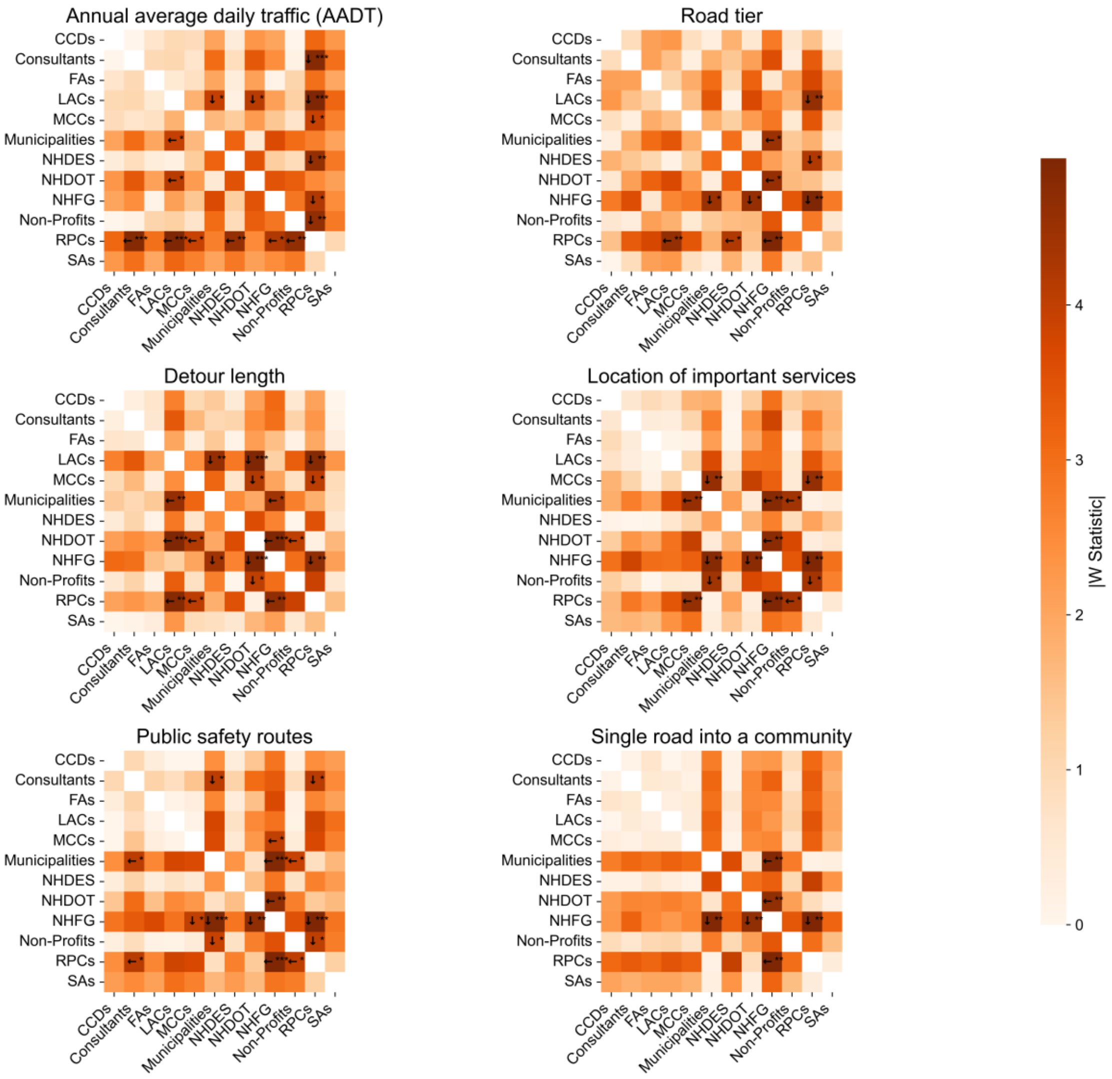

Figure S.10. Pairwise stakeholder differences in road-stream crossing (RSC) prioritization, based on the Dwass-Steel-Critchlow-Fligner (DSCF) post hoc test results for evaluation criteria of the ***road criticality*** *with significant Kruskal-Wallis results. The matrix displays the absolute value of the test statistic (|W|), where darker shades represent greater differences in prioritization between stakeholder groups. Arrows indicate the direction of higher ratings, identifying which group assigned greater importance to the goal. Statistical significance is marked by asterisks (* $p < 0.05$, ** $p < 0.01$, *** $p < 0.001$).*

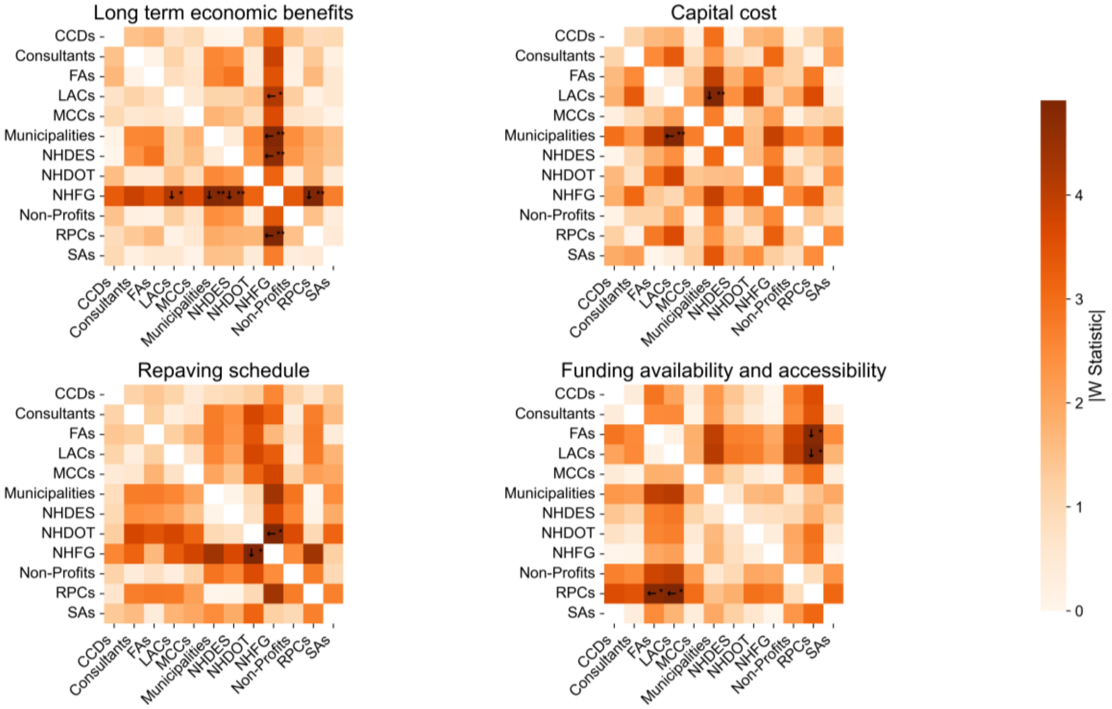

*Figure S.11. Pairwise stakeholder differences in road-stream crossing (RSC) prioritization, based on the Dwass-Steel-Critchlow-Fligner (DSCF) post hoc test results for evaluation criteria of the **economic impact** with significant Kruskal-Wallis results. The matrix displays the absolute value of the test statistic (|W|), where darker shades represent greater differences in prioritization between stakeholder groups. Arrows indicate the direction of higher ratings, identifying which group assigned greater importance to the goal. Statistical significance is marked by asterisks (* $p < 0.05$, ** $p < 0.01$, *** $p < 0.001$).*

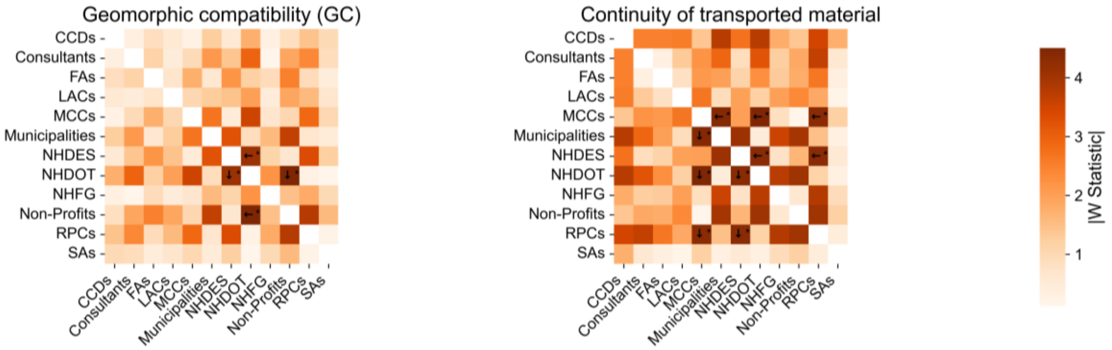

*Figure S.12. Pairwise stakeholder differences in road-stream crossing (RSC) prioritization, based on the Dwass-Steel-Critchlow-Fligner (DSCF) post hoc test results for evaluation criteria of the **environmental quality** with significant Kruskal-Wallis results. The matrix displays the absolute value of the test statistic (|W|), where darker shades represent greater differences in prioritization between stakeholder groups. Arrows indicate the direction of higher ratings, identifying which group assigned greater importance to the goal. Statistical significance is marked by asterisks (* $p < 0.05$, ** $p < 0.01$, *** $p < 0.001$).*

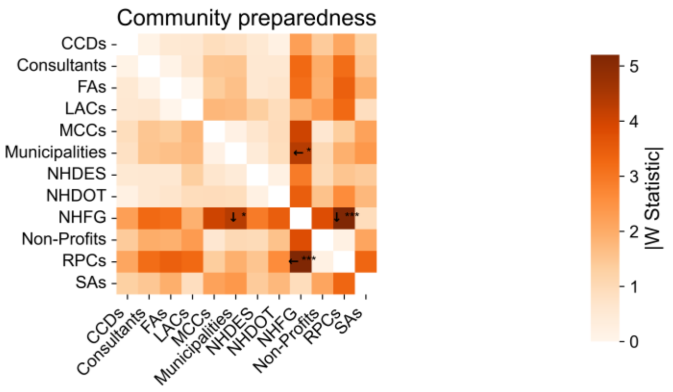

Figure S.13. Pairwise stakeholder differences in road-stream crossing (RSC) prioritization, based on the Dwass-Steel-Critchlow-Fligner (DSCF) post hoc test results for community preparedness. The matrix displays the absolute value of the test statistic (|W|), where darker shades represent greater differences in prioritization between stakeholder groups. Arrows indicate the direction of higher ratings, identifying which group assigned greater importance to the goal. Statistical significance is marked by asterisks (p < 0.05, ** p < 0.01, *** p < 0.001).*

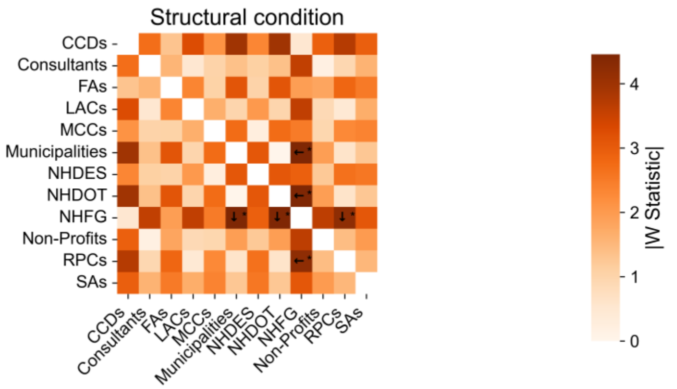

Figure S.14. Pairwise stakeholder differences in road-stream crossing (RSC) prioritization, based on the Dwass-Steel-Critchlow-Fligner (DSCF) post hoc test results for structural condition. The matrix displays the absolute value of the test statistic (|W|), where darker shades represent greater differences in prioritization between stakeholder groups. Arrows indicate the direction of higher ratings, identifying which group assigned greater importance to the goal. Statistical significance is marked by asterisks (p < 0.05, ** p < 0.01, *** p < 0.001).*

S5. Stakeholder-Informed Survey

The survey began with a consent form, followed by an introduction outlining the goals of the survey and definitions of the overarching goals and their associated evaluation criteria. Respondents were given explicit instructions to:

- Rate items from their professional standpoint, not personal preference
- Focus solely on the importance and relevance of each goal or criterion, without considering

data availability, quality, or feasibility
• Select “Unfamiliar” if they lacked knowledge of an item, or “Not related to the goal” if a criterion did not apply

Participants then provided their organizational role and relationship to stream-crossing management before rating the importance of the eight overarching goals. For each goal, a separate page was presented to rate the associated evaluation criteria (eight pages in total). The order of these pages was randomized to reduce potential bias. The survey concluded with open-ended questions to gather qualitative feedback. The full survey is provided below.

New Hampshire Stream Crossing Replacement Prioritization Survey

Survey Flow:

Standard: Consent (1 Question)
Standard: Introduction (5 Questions)
Standard: Overarching goals (10 Questions)

Block Randomizer: 8 -

- **Block: Wildlife Conservation and Restoration (15 Questions)**
- **Block: Road Criticality (8 Questions)**
- **Block: Environmental Quality (8 Questions)**
- **Block: Structural Risk (7 Questions)**
- **Block: Economic Impact (7 Questions)**
- **Block: Flood Vulnerability (7 Questions)**
- **Block: Community Support and Readiness (5 Questions)**
- **Block: Environmental Justice (4 Questions)**

Standard: Final section (6 Questions)

Page Break

Start of Block: Consent

CONSENT FORM FOR PARTICIPATION IN A RESEARCH STUDY

RESEARCHER AND TITLE OF STUDY

Koorosh Asadifakhr, PhD student, Civil and Environmental Engineering, University of New Hampshire
Weiwei Mo, Associate Professor, Civil and Environmental Engineering, University of New Hampshire
Our study is entitled: New Hampshire Stream Crossing Replacement Prioritization (UNH IRB-FY2023-154)

WHAT IS THE PURPOSE OF THIS FORM?

This consent form describes the research study and helps you to decide if you want to participate. It provides important information about what you will be asked to do in the study, about the risks and benefits of participating in the study, and about your rights as a research participant. You should:

- Read the information in this document carefully, and ask me or the research personnel any questions, particularly if you do not understand something.
- Not agree to participate until all your questions have been answered, or until you are sure that you want to.
- Understand that your participation in this study involves you completing a survey that will last about 30 minutes.
- Understand that the potential risks of participating in this study are minimal.

WHAT IS THE PURPOSE OF THIS STUDY?

This research is being conducted by the University of New Hampshire and funded by American Rescue Plan Act, and it aims to understand stakeholders' preferences related to stream crossing management criteria which would inform a framework to find win-win solutions for stream crossing management.

WHAT DOES YOUR PARTICIPATION IN THIS STUDY INVOLVE?

If you decide to participate in this study, you will complete a survey about your professional preferences related to stream crossing management criteria. The total amount of time should be no more than 30 minutes. Remember, you may stop at any time.

WHAT ARE THE POSSIBLE RISKS OF PARTICIPATING IN THIS STUDY?

Participating in this study involves minimal potential risk. While you will provide identifiable information, such as organization name and job role, we will de-identify all data before publication, ensuring minimal risk of confidentiality breaches with robust protections in place.

WHAT ARE THE POSSIBLE BENEFITS OF PARTICIPATING IN THIS STUDY?

There are no direct benefits of participating in this study. You are likely to benefit from learning various stream crossing management overarching goals and the evaluation criteria for each goal.

IF YOU CHOOSE TO PARTICIPATE IN THIS STUDY, WILL IT COST YOU ANYTHING?

There are no costs to you, other than your time, for being in this study.

WILL YOU RECEIVE ANY COMPENSATION FOR PARTICIPATING IN THIS STUDY?

You will not receive any compensation for participating in this study.

DO YOU HAVE TO TAKE PART IN THIS STUDY?

Taking part in this study is completely voluntary. You may choose not to take part at all. If you agree to participate, you may refuse to answer any question. If you decide not to participate, you will not be penalized or lose any benefits for which you would otherwise qualify.

CAN YOU WITHDRAW FROM THIS STUDY?

If you agree to participate in this study and you then change your mind, you may stop participating at any time. Any data collected as part of your participation will remain part of the study records. If you decide to stop participating at any time, you will not be penalized or lose any benefits for which you would otherwise qualify.

HOW WILL THE CONFIDENTIALITY OF YOUR RECORDS BE PROTECTED?

The confidentiality of all data and records associated with your participation in this research will be actively protected. Further, any communication via the Internet poses minimal risk of a breach of confidentiality. To help protect the confidentiality of your information, we are storing the data on USNH IT secure cloud storage. Koorosh Asadifakhr and Drs. Weiwei Mo and Erin Bell from the University of New Hampshire will have access to the data, but there are rare circumstances under which others may have access to data. Identifiable information will not be shared with a third-party data processor (e.g., transcription services). Moreover, identifiable information will not be disclosed, and direct quotes will not be reported. The data, once de-identified, may be published alongside the results (e.g., in aggregate, using pseudonyms) in journal papers.

WHOM TO CONTACT IF YOU HAVE QUESTIONS ABOUT THIS STUDY

If you have any questions pertaining to the research, you can contact Koorosh Asadifakhr at Koorosh.Asadifakhr@unh.edu or the principal investigator, Dr. Weiwei Mo, at telephone: (603) 862-2808 or email: Weiwei.Mo@unh.edu to discuss them.

If you have questions about your rights as a research subject you can contact Melissa McGee in UNH Research Integrity Services, 603/862-2005 or melissa.mcgee@unh.edu to discuss them.

- Click the next page button if you consent to participate in the research study.
- Click the close tab/window button if you decline to participate in the research study.

End of Block: Consent

Start of Block: Introduction

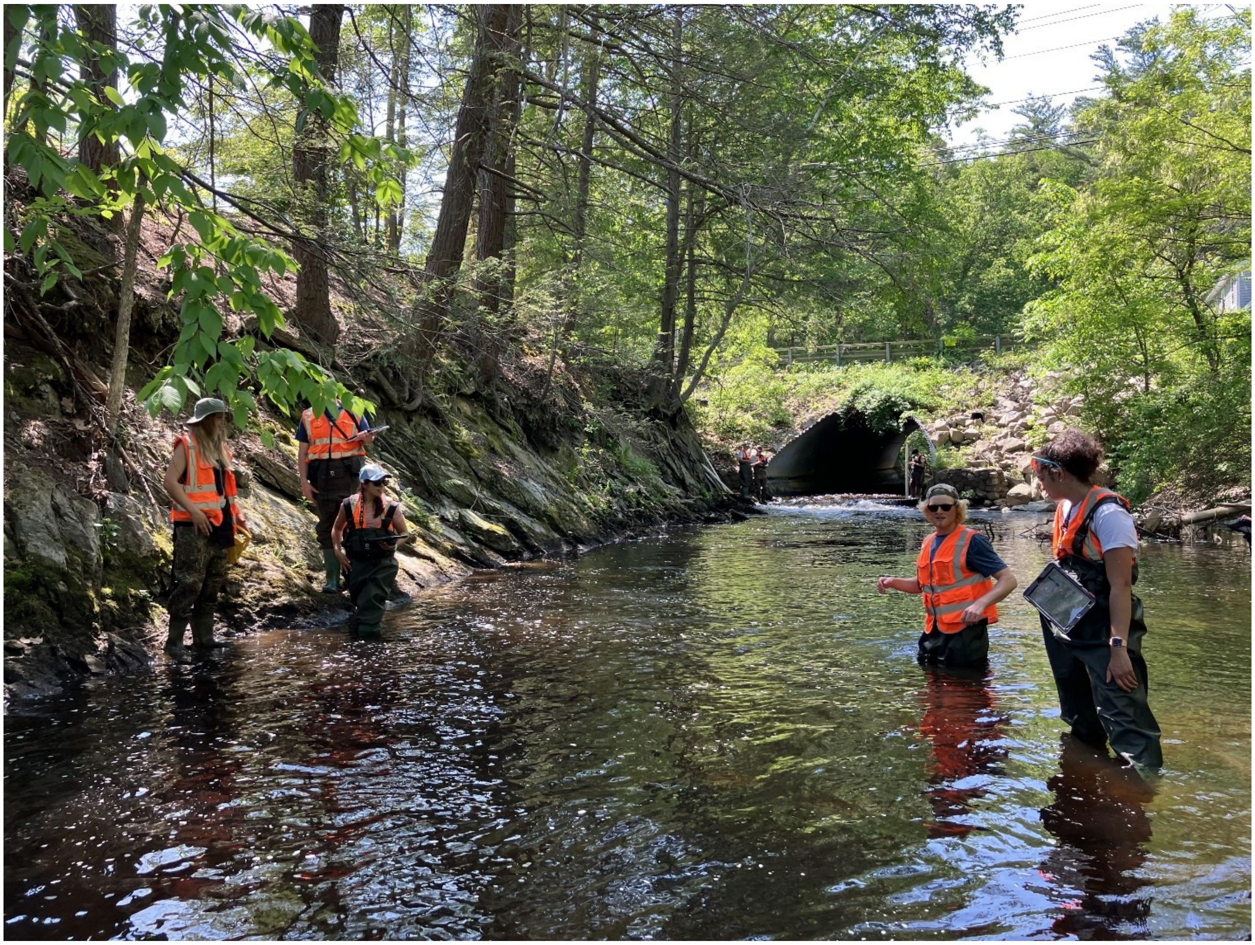

Thank you for your willingness to participate in the New Hampshire Stream Crossing Replacement Prioritization Survey. Please read this brief information carefully before you begin the survey.

Stream crossings are structures (for instance, culverts, bridges, and arches) that allow roads to pass over water bodies, potentially impacting human and wildlife communities. Approximately 20,000 such crossings exist within New Hampshire. Many of these crossings are aged or failed, exacerbating stream fragmentation and flood risks, yet resources to manage these culverts are often limited and scattered across stakeholders. Therefore, it is vital to understand the priorities and interests of diverse stakeholders and develop optimal management scenarios. To achieve this, we are engaging stakeholders in stream crossing prioritization.

We identified eight **overarching goals** and their respective **evaluation criteria** for stream crossing replacement prioritization through a review of scientific journals and project reports and using experts' inputs. Each **overarching goal** represents a strategic aspiration addressing a specific aspect of stream crossing management to achieve optimal ecological, economic, and societal outcomes, and **evaluation criteria** describe specific measurable or observable characteristics of a goal. If a criterion pertains to multiple overarching goals, it is accounted for

in each relevant goal. This survey intends to broadly elicit stakeholders' weighting and feedback on the overarching goals and their corresponding evaluation criteria to inform a prioritization framework that will facilitate the identification of win-win stream crossing management strategies. Your time and collaboration are appreciated.

SURVEY INSTRUCTIONS

In this survey, we will ask you to rate the importance of **overarching goals** and their **evaluation criteria**. If you are not familiar with a goal or criterion, you can choose the "Unfamiliar" choice. Moreover, for the evaluation criteria, if you think a criterion does not belong to a goal, you can choose the "Not related to the goal" choice. **It is important to rate the goals and their criteria based on your job/organization role, not your personal preferences, and focus solely on their significance and relevance without taking the feasibility of data acquisition or data quality into consideration.**

Page Break

Which of the following best describes your organization type?

▼ Federal agency (1) ... Other (8)

Display This Question:

If Which of the following best describes your organization type? = Other

Please specify your organization type.

____

Please enter the name of your organization and your job title (or role).

____

Please describe how your work is related to stream crossing management.

____

____

____

____

____

End of Block: Introduction

Start of Block: Overarching goals

Overarching Goals
These eight **overarching goals** represent strategic aspirations addressing specific aspects of stream crossing management to achieve optimal ecological, economic, and societal outcomes. Please submit the level of importance your organization assigns to the following overarching goals**. It is important to rate these goals based on your job/organization role, not your personal preferences, and focus solely on their significance and relevance without taking the feasibility of data acquisition or data quality into consideration.**

JS

Wildlife Conservation and Restoration
Click here to see the description
This goal focuses on the compatibility of fish and wildlife to pass through a stream crossing structure as well as the greatest opportunities to restore habitat connectivity within a riverine system.

- ◯ Not at all important
- ◯ Slightly important
- ◯ Moderately important
- ◯ Very important
- ◯ Extremely important
- ◯ Unfamiliar

JS

Road Criticality
Click here to see the description

This goal focuses on the community importance of the road segment and stream crossing structure to the functional operation of the transportation system.

- Not at all important
- Slightly important
- Moderately important
- Very important
- Extremely important
- Unfamiliar

JS

Environmental Quality
Click here to see the description
This goal focuses on the existing water quality of the stream segment and whether the stream crossing structure negatively impacts water quality.

- Not at all important
- Slightly important
- Moderately important
- Very important
- Extremely important
- Unfamiliar

Flood Vulnerability
Click here to see the description
This goal evaluates the compatibility of the stream crossing structure to accommodate the natural shape of the river and transport flood flows, as well as other evidence of flood risk.

- ◯ Not at all important
- ◯ Slightly important
- ◯ Moderately important
- ◯ Very important
- ◯ Extremely important
- ◯ Unfamiliar

Economic Impact
Click here to see the description
The long- and short-term financial considerations of stream crossing replacement.

- ◯ Not at all important
- ◯ Slightly important
- ◯ Moderately important
- ◯ Very important
- ◯ Extremely important
- ◯ Unfamiliar

JS

Structural Risk
Click here to see the description
The condition of the stream crossing structure and other structural integrity factors that affect stream crossing risk and magnitude of impact.

- ◯ Not at all important
- ◯ Slightly important
- ◯ Moderately important
- ◯ Very important
- ◯ Extremely important
- ◯ Unfamiliar

Community Support and Readiness
Click here to see the description
How well a community is prepared for and supportive of stream crossing management.

- ◯ Not at all important
- ◯ Slightly important
- ◯ Moderately important
- ◯ Very important
- ◯ Extremely important
- ◯ Unfamiliar

Environmental Justice
Click here to see the description
This goal ensures equitable distribution of resources and benefits among communities

(distributional equity), and fosters inclusive, fair decision-making procedures (procedural equity).

- ○ Not at all important
- ○ Slightly important
- ○ Moderately important
- ○ Very important
- ○ Extremely important
- ○ Unfamiliar

Do you have any recommendations for the overarching goals, or any additional goals to suggest? Please provide details below.

____

End of Block: Overarching goals

Start of Block: Wildlife Conservation and Restoration

Wildlife Conservation and Restoration focuses on the compatibility of fish and wildlife to pass through a stream crossing structure as well as the greatest opportunities to restore habitat connectivity within a riverine system.

Please submit the importance of each criterion when you assess stream crossings' performance in terms of wildlife conservation and restoration. **It is important to rate the criteria below based on your job/organization role, not your personal preferences, and focus solely on their significance and relevance without taking the feasibility of data acquisition or data quality into consideration.**

Aquatic organism passage (AOP)
Click here to see the description
The ability if the stream crossing to allow for aquatic organism passage (AOP) (i.e. passability). AOP is used to identify crossings that may be barriers to aquatic organisms and prohibit their movement through the stream.

- Not at all important
- Slightly important
- Moderately important
- Very important
- Extremely important
- Not related to the goal
- Unfamiliar (25)

Watershed AOP
Click here to see the description
Considers the upstream and downstream organism movement blockages of a stream crossing. Upstream and downstream blockages determine whether managing the current

stream crossing can be effective for restoring sea-run fish species. It views stream crossing management from a whole watershed scale.

- ◯ Not at all important
- ◯ Slightly important
- ◯ Moderately important
- ◯ Very important
- ◯ Extremely important
- ◯ Not related to the goal
- ◯ Unfamiliar (25)

JS

Terrestrial organism passage
Click here to see the description

The ability if the stream crossing to allow for terrestrial organisms (e.g., mammals, reptiles, etc.) to pass.

- Not at all important
- Slightly important
- Moderately important
- Very important
- Extremely important
- Not related to the goal
- Unfamiliar (25)

JS

Habitat quantity
Click here to see the description
The amount of upstream and downstream reconnected habitat when a stream crossing is

replaced. Calculated from the replaced stream crossing to known natural or man-made barriers upstream and downstream.

- Not at all important
- Slightly important
- Moderately important
- Very important
- Extremely important
- Not related to the goal
- Unfamiliar (25)

Habitat quality
Click here to see the description

The condition and ecological suitability of a stream environment, determined by factors such as water quality, stream flow, and the life cycle of aquatic organisms.

- ○ Not at all important
- ○ Slightly important
- ○ Moderately important
- ○ Very important
- ○ Extremely important
- ○ Not related to the goal
- ○ Unfamiliar (25)

JS

Downstream and upstream blockage rate
Click here to see the description

The downstream and upstream blockage rate is calculated based on the lowest aquatic organism passability of downstream and upstream barriers.

- ○ Not at all important
- ○ Slightly important
- ○ Moderately important
- ○ Very important
- ○ Extremely important
- ○ Not related to the goal
- ○ Unfamiliar (25)

Number of barriers
Click here to see the description
Number of upstream and/or downstream barriers.

- ◯ Not at all important
- ◯ Slightly important
- ◯ Moderately important
- ◯ Very important
- ◯ Extremely important
- ◯ Not related to the goal
- ◯ Unfamiliar (25)

JS

Conservation status
Click here to see the description
Conservation status of lands within the upstream watershed, and up- and downstream

buffer. It estimates the degree to which watershed and sub-watershed buffers were conserved as a way to estimate the potential for habitat to change over time.

- Not at all important
- Slightly important
- Moderately important
- Very important
- Extremely important
- Not related to the goal
- Unfamiliar (25)

Wetland connectivity
Click here to see the description
The degree of wetland re-connection gained.

- ◯ Not at all important
- ◯ Slightly important
- ◯ Moderately important
- ◯ Very important
- ◯ Extremely important
- ◯ Not related to the goal
- ◯ Unfamiliar (25)

JS

Stream order
Click here to see the description

A positive whole number used in geomorphology and hydrology to indicate the level of branching in a river system.

- ◯ Not at all important
- ◯ Slightly important
- ◯ Moderately important
- ◯ Very important
- ◯ Extremely important
- ◯ Not related to the goal
- ◯ Unfamiliar (25)

JS

Risk of wildlife collision
Click here to see the description
Risk of Wildlife-vehicle collisions within a certain distance from the stream crossing.

- ◯ Not at all important
- ◯ Slightly important
- ◯ Moderately important
- ◯ Very important
- ◯ Extremely important
- ◯ Not related to the goal
- ◯ Unfamiliar (25)

JS

Special species presence and population
Click here to see the description

Whether a special species is present in the stream and its population (e.g., endangered or not), e.g., eastern brook trout.

- ○ Not at all important
- ○ Slightly important
- ○ Moderately important
- ○ Very important
- ○ Extremely important
- ○ Not related to the goal
- ○ Unfamiliar (25)

JS

Species diversity
Click here to see the description
The number of species present in the stream/watershed.

- ○ Not at all important
- ○ Slightly important
- ○ Moderately important
- ○ Very important
- ○ Extremely important
- ○ Not related to the goal
- ○ Unfamiliar (25)

Do you have any recommendations for the criteria associated with **Wildlife Conservation and Restoration**, or any additional criteria to suggest? Please provide details below.

____

End of Block: Wildlife Conservation and Restoration

Start of Block: Road Criticality

Road Criticality focuses on the community importance of the road segment and stream crossing structure to the functional operation of the transportation system.

Please submit the importance of each criterion when you assess stream crossings' performance in terms of road criticality. **It is important to rate the criteria below based on your job/organization role, not your personal preferences, and focus solely on their significance**

and relevance without taking the feasibility of data acquisition or data quality into consideration.

Annual average daily traffic (AADT)
Click here to see the description
Average daily traffic on a roadway link for all days of the week during a period of one year, expressed in VPD (vehicles per day).

- ◯ Not at all important
- ◯ Slightly important
- ◯ Moderately important
- ◯ Very important
- ◯ Extremely important
- ◯ Not related to the goal (25)
- ◯ Unfamiliar

Road tier
Click here to see the description
Tier 1 – Interstates, Turnpikes, and Divided Highways; Tier 2 – Statewide Corridors; Tier

3 – Regional Transportation Corridors; Tier 4 – Local Connectors; Tier 5 – Local Roads; Tier 6 – Off Network

- Not at all important
- Slightly important
- Moderately important
- Very important
- Extremely important
- Not related to the goal (25)
- Unfamiliar

JS

Detour length
Click here to see the description

The total additional travel a through-bound vehicle would experience if a stream crossing fails.

- ◯ Not at all important
- ◯ Slightly important
- ◯ Moderately important
- ◯ Very important
- ◯ Extremely important
- ◯ Not related to the goal (25)
- ◯ Unfamiliar

JS

Location of important services
Click here to see the description

Stream crossing's distance to the location of important services, such as hospitals, fire departments, police, etc.

- ◯ Not at all important
- ◯ Slightly important
- ◯ Moderately important
- ◯ Very important
- ◯ Extremely important
- ◯ Not related to the goal (25)
- ◯ Unfamiliar

Public safety routes
Click here to see the description

Whether the stream crossing is on the routes of emergency evacuation or buses/school buses.

- ◯ Not at all important
- ◯ Slightly important
- ◯ Moderately important
- ◯ Very important
- ◯ Extremely important
- ◯ Not related to the goal (25)
- ◯ Unfamiliar

JS

Single road into a community
Click here to see the description
Whether the stream crossing is on a single road going in and out of the community.

- ○ Not at all important
- ○ Slightly important
- ○ Moderately important
- ○ Very important
- ○ Extremely important
- ○ Not related to the goal (25)
- ○ Unfamiliar

Do you have any recommendations for the criteria associated with **Road Criticality**, or any additional criteria to suggest? Please provide details below.

____

End of Block: Road Criticality

Start of Block: Environmental Quality

Environmental Quality focuses on the existing water quality of the stream segment and whether the stream crossing structure negatively impacts water quality.

Please submit the importance of each criterion when you assess stream crossings' performance in terms of environmental quality. **It is important to rate the criteria below based on your job/organization role, not your personal preferences, and focus solely on their significance**

and relevance without taking the feasibility of data acquisition or data quality into consideration.

JS

Geomorphic compatibility (GC)
Click here to see the description
The Geomorphic Compatibility (GC) score evaluates how well the stream crossing structure fits within the natural shape and form of the stream and whether it alters water and sediment transport. This is completed to predict the long-term compatibility of a stream crossing with river channel form. The GC score is derived from a model that uses the survey data to rank the crossings from “fully compatible” to “fully incompatible”.

- ◯ Not at all important
- ◯ Slightly important
- ◯ Moderately important
- ◯ Very important
- ◯ Extremely important
- ◯ Not related to the goal (25)
- ◯ Unfamiliar

JS

Continuity of sediment, carbon, nutrients, large wood, and other transport constituent
Click here to see the description
Uninterrupted and stable transport and distribution of these elements through a riverine

system, contributing to habitat formation, nutrient cycling, carbon sequestration, and overall ecosystem functioning and resilience.

- ◯ Not at all important
- ◯ Slightly important
- ◯ Moderately important
- ◯ Very important
- ◯ Extremely important
- ◯ Not related to the goal (25)
- ◯ Unfamiliar

JS

Erosion
Click here to see the description

The removal of sediment from around or beneath a stream crossing, as well as upstream or downstream due to the flow of water.

- ○ Not at all important
- ○ Slightly important
- ○ Moderately important
- ○ Very important
- ○ Extremely important
- ○ Not related to the goal (25)
- ○ Unfamiliar

JS

Water use
Click here to see the description

Whether the water is used as a source for public drinking water supply or used for recharging groundwater aquifers.

- Not at all important
- Slightly important
- Moderately important
- Very important
- Extremely important
- Not related to the goal (25)
- Unfamiliar

Water quality impairment
Click here to see the description
Indicates if the stream crossing is on an impaired water body.

- ○ Not at all important
- ○ Slightly important
- ○ Moderately important
- ○ Very important
- ○ Extremely important
- ○ Not related to the goal (25)
- ○ Unfamiliar

JS

Entrenchment ratio
Click here to see the description
Calculated as the flood-prone width divided by the bankfull width. Entrenchment ratio is the vertical containment of a river as seen by the relationship between the channel (within

the bankfull width) and the surrounding floodplain (within the flood prone width). The lower the ratio, the more entrenched a channel is.

○ Not at all important

○ Slightly important

○ Moderately important

○ Very important

○ Extremely important

○ Not related to the goal (25)

○ Unfamiliar

Do you have any recommendations for the criteria associated with **Environmental Quality**, or any additional criteria to suggest? Please provide details below.

____

End of Block: Environmental Quality

Start of Block: Structural Risk

Structural Risk focuses on the condition of the stream crossing structure and other structural integrity factors that affect stream crossing risk and magnitude of impact.

Please submit the importance of each criterion when you assess stream crossings' performance in terms of structural risk. **It is important to rate the criteria below based on your job/organization role, not your personal preferences, and focus solely on their significance**

and relevance without taking the feasibility of data acquisition or data quality into consideration.

Structural condition
Click here to see the description
The physical state of a stream crossing encompasses its material integrity, overall stability, and ability to safely convey stream flows without compromising its structure or the infrastructure it supports.

- ○ Not at all important
- ○ Slightly important
- ○ Moderately important
- ○ Very important
- ○ Extremely important
- ○ Not related to the goal (25)
- ○ Unfamiliar

Age of the stream crossing
Click here to see the description
Age of the stream crossing

- ◯ Not at all important
- ◯ Slightly important
- ◯ Moderately important
- ◯ Very important
- ◯ Extremely important
- ◯ Not related to the goal (25)
- ◯ Unfamiliar

JS

Material
Click here to see the description
Material of the stream crossing structure.

- ◯ Not at all important
- ◯ Slightly important
- ◯ Moderately important
- ◯ Very important
- ◯ Extremely important
- ◯ Not related to the goal (25)
- ◯ Unfamiliar

JS

Size and depth
Click here to see the description
Attributes of a stream crossing (depth of cover and cross-sectional area) that may influence

the magnitude of a stream crossing replacement project as well as the impact of failure to public safety.

- ○ Not at all important
- ○ Slightly important
- ○ Moderately important
- ○ Very important
- ○ Extremely important
- ○ Not related to the goal (25)
- ○ Unfamiliar

JS

Stream fit
Click here to see the description
How well the stream crossing structure fits within the natural shape and form of the stream

channel (percent bankfull width) and whether flow (hydraulic capacity) could negatively impact the structure, itself.

- ○ Not at all important
- ○ Slightly important
- ○ Moderately important
- ○ Very important
- ○ Extremely important
- ○ Not related to the goal (25)
- ○ Unfamiliar

Do you have any recommendations for the criteria associated with **Structural Risk**, or any additional criteria to suggest? Please provide details below.

____

End of Block: Structural Risk

Start of Block: Economic Impact

Economic Impact focuses on the long- and short-term financial considerations of stream crossing maintenance and/or replacement.

Please submit the importance of each criterion when you assess stream crossings' performance in terms of economic impact. **It is important to rate the criteria below based on your job/organization role, not your personal preferences, and focus solely on their significance**

and relevance without taking the feasibility of data acquisition or data quality into consideration.

JS

Long term economic benefits
Click here to see the description
Long term economic benefits to the local communities reflected through reduced flooding risk, better environmental quality, higher property value, better tourism routes, etc.

- Not at all important
- Slightly important
- Moderately important
- Very important
- Extremely important
- Not related to the goal (25)
- Unfamiliar

JS

Capital cost
Click here to see the description

The capital cost refers to the estimated total expense incurred in acquiring, constructing, or upgrading a stream crossing.

- ◯ Not at all important
- ◯ Slightly important
- ◯ Moderately important
- ◯ Very important
- ◯ Extremely important
- ◯ Not related to the goal (25)
- ◯ Unfamiliar

JS

O&M cost
Click here to see the description

The O&M cost refers to the annual recurring cost for operating and maintaining the stream crossing.

- Not at all important
- Slightly important
- Moderately important
- Very important
- Extremely important
- Not related to the goal (25)
- Unfamiliar

JS

Repaving schedule
Click here to see the description

Indicates if the stream crossing on a road scheduled to be repaved in the future, such as the next 5, 10, 15 years.

- ○ Not at all important
- ○ Slightly important
- ○ Moderately important
- ○ Very important
- ○ Extremely important
- ○ Not related to the goal (25)
- ○ Unfamiliar

JS

Funding availability and accessibility
Click here to see the description
The ease and availability of financial resources or capital for individuals, organizations, or

projects. It encompasses the extent to which funding options and opportunities are accessible, inclusive, and readily obtainable by diverse individuals or groups.

- ○ Not at all important
- ○ Slightly important
- ○ Moderately important
- ○ Very important
- ○ Extremely important
- ○ Not related to the goal (25)
- ○ Unfamiliar

Do you have any recommendations for the criteria associated with **Economic Impact**, or any additional criteria to suggest? Please provide details below.

____

End of Block: Economic Impact

Start of Block: Flood Vulnerability

Flood Vulnerability evaluates the compatibility of the stream crossing structure to accommodate the natural shape of the river and transport flood flows, as well as other evidence of flood risk.

Please submit the importance of each criterion when you assess stream crossings' performance in terms of flood vulnerability. **It is important to rate the criteria below based on your job/organization role, not your personal preferences, and focus solely on their significance**

and relevance without taking the feasibility of data acquisition or data quality into consideration.

Hydraulic capacity
Click here to see the description
Predicts how a stream crossing will transport flows during storm events. This information is helpful to make mindful decisions on flood probability prediction and identifying the most vulnerable infrastructure.

- ○ Not at all important
- ○ Slightly important
- ○ Moderately important
- ○ Very important
- ○ Extremely important
- ○ Not related to the goal (25)
- ○ Unfamiliar

Geomorphic compatibility (GC)
Click here to see the description
The Geomorphic Compatibility (GC) score evaluates how well the stream crossing structure fits within the natural shape and form of the stream and whether it alters water and sediment transport. This is completed to predict the long-term compatibility of a

stream crossing with river channel form. The GC score is derived from a model that uses the survey data to rank the crossings from "fully compatible" to "fully incompatible".

- Not at all important
- Slightly important
- Moderately important
- Very important
- Extremely important
- Not related to the goal (25)
- Unfamiliar

JS

Flood days per year
Click here to see the description

Estimated number of days where water overtops the road surface during an average years' worth of rainfall.

- ○ Not at all important
- ○ Slightly important
- ○ Moderately important
- ○ Very important
- ○ Extremely important
- ○ Not related to the goal (25)
- ○ Unfamiliar

Documented history of flooding
Click here to see the description
Whether there has been any documented history of the culvert being washed out/flooded.

- ◯ Not at all important
- ◯ Slightly important
- ◯ Moderately important
- ◯ Very important
- ◯ Extremely important
- ◯ Not related to the goal (25)
- ◯ Unfamiliar

JS

Climate resiliency
Click here to see the description

Changes in the occurrence, magnitude, and characteristics of flooding events due to climate change and how climate change may influence evacuation during a flooding event.

- ○ Not at all important
- ○ Slightly important
- ○ Moderately important
- ○ Very important
- ○ Extremely important
- ○ Not related to the goal (25)
- ○ Unfamiliar

Do you have any recommendations for the criteria associated with **Flood Vulnerability**, or any additional criteria to suggest? Please provide details below.

____

End of Block: Flood Vulnerability

Start of Block: Community Support and Readiness

Community Support and Readiness how well a community is prepared for and supportive of stream crossing management.

Please submit the importance of each criterion when you assess stream crossings' performance in terms of community support and readiness. **It is important to rate the criteria below based on your job/organization role, not your personal preferences, and focus solely on their**

significance and relevance without taking the feasibility of data acquisition or data quality into consideration.

JS

Community preparedness
Click here to see the description
Whether the stream crossing is referenced in Master Plans, flood hazard database, municipal planning documents such as Hazard, Master, or Capital Improvement Plans, Regional planning documents such as Transportation, Corridor, or Regional Plans.

- ○ Not at all important
- ○ Slightly important
- ○ Moderately important
- ○ Very important
- ○ Extremely important
- ○ Not related to the goal (25)
- ○ Unfamiliar

JS

State and federal support
Click here to see the description

Stream crossings or areas that contain stream crossings that have been identified by state and/or federal agencies as a priority.

- Not at all important
- Slightly important
- Moderately important
- Very important
- Extremely important
- Not related to the goal (25)
- Unfamiliar

JS

Funding availability and accessibility
Click here to see the description
The ease and availability of financial resources or capital for individuals, organizations, or

projects. It encompasses the extent to which funding options and opportunities are accessible, inclusive, and readily obtainable by diverse individuals or groups.

○ Not at all important

○ Slightly important

○ Moderately important

○ Very important

○ Extremely important

○ Not related to the goal (25)

○ Unfamiliar

Do you have any recommendations for the criteria associated with **Community Support and Readiness**, or any additional criteria to suggest? Please provide details below.

____

End of Block: Community Support and Readiness

Start of Block: Environmental Justice

Environmental Justice ensures equitable distribution of resources and benefits among communities (distributional equity), and fosters inclusive, fair decision-making procedures (procedural equity).

Please submit the importance of each criterion when you assess stream crossings' performance in terms of environmental justice. **It is important to rate the criteria below based on your job/organization role, not your personal preferences, and focus solely on their significance**

and relevance without taking the feasibility of data acquisition or data quality into consideration.

JS

Distributional equity
Click here to see the description
Fairness and equality in the distribution of resources for stream crossing replacement, benefits gained from stream crossing replacement, or management burdens among individuals or groups within a society.

- ○ Not at all important
- ○ Slightly important
- ○ Moderately important
- ○ Very important
- ○ Extremely important
- ○ Not related to the goal (25)
- ○ Unfamiliar

JS

Procedural equity
Click here to see the description
Ensuring that the stream crossing replacement decision-making and implementation

processes employed are inclusive, unbiased, consistent, and provide individuals with a sense of fairness and legitimacy.

- ○ Not at all important
- ○ Slightly important
- ○ Moderately important
- ○ Very important
- ○ Extremely important
- ○ Not related to the goal (25)
- ○ Unfamiliar

Do you have any recommendations for the criteria associated with **Environmental Justice**, or any additional criteria to suggest? Please provide details below.

____

End of Block: Environmental Justice

Start of Block: Final section

Data Sources, Final Thoughts, and Additional Information
Thank you so much for rating our overarching goals and their evaluation criteria. Please kindly respond to the following questions.

Would you kindly provide any data sources and/or references that can be used to quantify the evaluation criteria mentioned in previous sections? Please be as detailed as possible, provide links to information, etc.

Which organizations, technical experts, or other entities do you collaborate with, if any, for prioritizing and replacing stream crossings?

Besides funding, what are the biggest challenges you see in stream crossing replacement prioritization in New Hampshire?

What tools, data, and/or resources (besides funding) would help facilitate stream crossing prioritization and replacement in your role?

____

____

____

____

____

If you have any general comments and suggestions to improve this study or this survey, please provide here.

____

____

____

____

____

End of Block: Final section
